# Diaminomaleonitrile (DAMN)-Based Salen-Type Cobalt Complexes as Cyclable Sensors for H_2_S/HS^–^: Biological Applications

**DOI:** 10.1021/acs.inorgchem.5c01186

**Published:** 2025-05-13

**Authors:** Alessio Trerotola, Viktoriia Vykhovanets, Tonino Caruso, Daniela Guarnieri, Marina Lamberti, Stefano Milione, Maria Strianese

**Affiliations:** Dipartimento di Chimica e Biologia “Adolfo Zambelli” and INSTM Research Unit, 19028 Università degli Studi di Salerno, Via Giovanni Paolo II, 132, Fisciano, SA 84084, Italy

## Abstract

In the current contribution, the synthesis and characterization of new diaminomaleonitrile-based salen cobalt complexes *L*_1_Co and *L*_2_Co (*L*_1_-H = 2,3-bis­[[(2,4-dihydroxy-phenyl)­(methylene)]­amino]-2-butenedinitrile, *L*_2_-H = 2,3-bis­[[(2-hydroxy-4-(diethylamino)­phenyl)­(methylene)]­amino]-2-butenedinitrile) are reported. Structural analysis and electronic properties of the complexes were assessed, providing a basis for their application as H_2_S sensors. Spectroscopic investigations revealed an enhancement in the fluorescence intensities upon H_2_S addition. A reversible fluorescence response was observed when the systems were shifted from H_2_S-rich to oxygenated environments, demonstrating their reusability and adaptability. To further establish the mechanism of interaction of the HS^–^ with the cobalt complexes, electrochemical analysis was conducted, providing evidence of HS^–^ coordination to the cobalt centers. Moreover, biological assessment of the sensors was performed in HepG2 cells, revealing their low cytotoxicity and efficient cell permeability. Fluorescence imaging experiments provided evidence of intracellular detection of exogenous H_2_S. The reversibility of the sensing mechanism was confirmed in living cells: subsequent addition of HS^–^ and oxygen-saturated buffer to the cells tunes their fluorescence accordingly, in a cyclable manner, as in the experiments performed *in vitro*. This study provides evidence that cobalt-based DAMN complexes work as robust, reversible, and selective detection systems for H_2_S, demonstrating their suitability for applications in both biological and environmental contexts.

## Graphical abstract

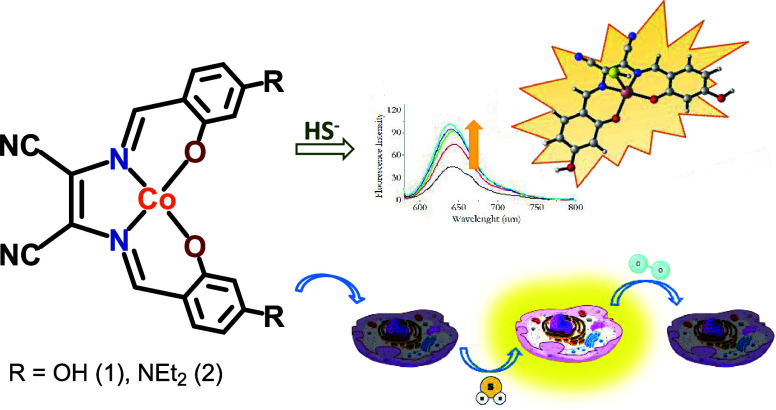

## Introduction

The traditional image of H_2_S as a toxic molecule began to change at the end of the 20th century when the pioneering studies of Abe and Kimura^1–3^ demonstrated the existence of pathways for endogenous generation of H_2_S and sulfur metabolism. This discovery may not be surprising if thinking that natural sulfur-rich water was used in the SPA for treatments of skin already in ancient times and nowadays.^4^

By now, in the scientific community, it is commonly acknowledged that H_2_S is an important biological molecule that plays key roles in various disciplines,^5,6^ spanning from biomedicine to environmental chemistry. A common need across these different disciplines is the possibility of detecting and measuring H_2_S in complex contexts. A shared approach for H_2_S detection makes use of activity-based fluorescent probes, many of which can be easily set up or are commercially available.^7,8^ These probes function by exploiting a chemical modification of the sensing molecule promoted by H_2_S, which results in a fluorescent response.

Although different fluorescence-based H_2_S detecting systems have been devised,^9–14^ reversible systems that function in complex environments, such as live cells or tissues, remain a challenging and unmet task.^7^

The chemical reactions used in these fluorescent probes have been based on H_2_S-mediated reduction, addition to electrophiles, or metal precipitation.^9,10^ The coordination reaction of the sulfide anion to the metal centers has been less explored notwithstanding that this is a particularly promising approach to develop reusable probes as the coordination can be reversible and the sulfide anion can be opportunely removed from the metal center to restore the initial metal complex.^14^

One of the first reversible sensors for H_2_S detection was reported in 2011: the HS^–^-sensing molecule was a mixed-valence diruthenium complex [Ru_2_TIEDCl_4_] (TIED = tetra-imino-ethylene-dimacrocycle, Chart 1, a), which exhibits an unpaired electron completely delocalized on the whole structure and for which authors claim selectivity and reversibility in HS^–^ recognition.^15^ In 2014, Pluth and co-workers reported that cobalt­(II) and zinc phtalocyanines react with H_2_S and can be successfully used as sensing devices for H_2_S detection in a reversible manner (Chart 1, b).^16^ For the cobalt system, reversibility is ensured via subsequent cycles of reduction and oxidation of the metal center whereas the zinc-phtalocyanine analogue was found effective as a reversible H_2_S-detecting system by the coordination-based mechanism. Upon coordination of HS^–^ to the zinc center, a suitable proton source (e.g., a weak acid) could remove the analyte from the metal center.^16^ Both the mixed-valence diruthenium complex and the metal phtalocyanines were assayed by UV–vis spectroscopy measurements *in vitro*.^15,16^

**Chart 1:**
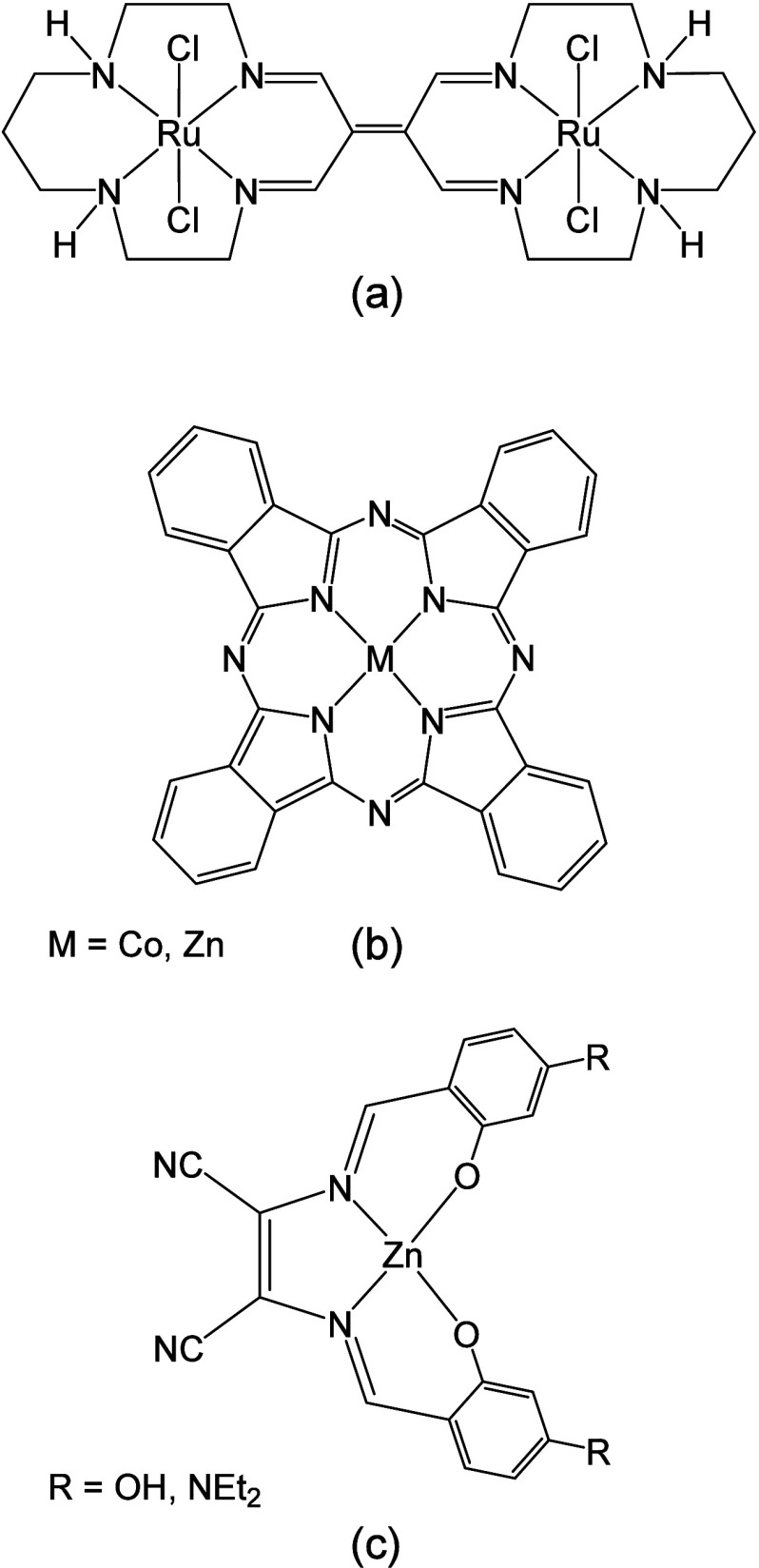
Reversible H_2_S sensors in the literature.

More recently, we and others proposed zinc-salen complexes and hemoglobin as reversible fluorescence-based H_2_S-detecting systems and again they were mainly tested for *in vitro* experiments.^9,14,17–22^

Cobalt-based coordination complexes have a great potential to act as fluorescent probes for H_2_S as the cobalt ion is a redox-active and “borderline” soft Lewis acid with a strong affinity for sulfur. Despite these, the number of cobalt complexes used at this scope is very limited and the most notable example remains that reported by Pluth in 2014. In the framework of our research interests, a few years ago, we reported diaminomalonitrile (DAMN)-based salen zinc complexes as successful H_2_S sensors.^18^ The electron donor (−OH or −NEt_2_ group) and the electron acceptor (−C=N group) present on the ligand skeleton confer interesting optical behaviors to the corresponding zinc complexes (Chart 1, c) that effectively act in the colorimetric and fluorescence detection of HS^–^.

In this contribution, we thought of expanding the use of these colorful salen ligands to develop new fluorescent cobalt-based probes. Below, the synthesis and characterization of two new DAMN-based complexes and their application in the sensing of H_2_S is reported. Experiments performed in living cells result in reversible responses, providing proof of principle for the cyclability of the systems under investigation in such complex environments.

## Results and Discussion

The Co complexes synthesized and studied in this work are displayed in Scheme 1. They were synthesized at room temperature by reacting the neutral proligands^18^ 2,3-bis­[[(2,4-dihydroxy-phenyl)­(methylene)]­amino]-2-butenedinitrile (*L*_1_-H) and 2,3-bis­[[(2-hydroxy-4-(diethylamino)­phenyl)­(methylene)]­amino]-2-butenedinitrile (*L*_2_-H) with cobalt acetate in methanol, by following a modified literature procedure,^23^ and were isolated as dark-red and dark-green solid powders in high yields (see Scheme 1).

**Scheme 1:**
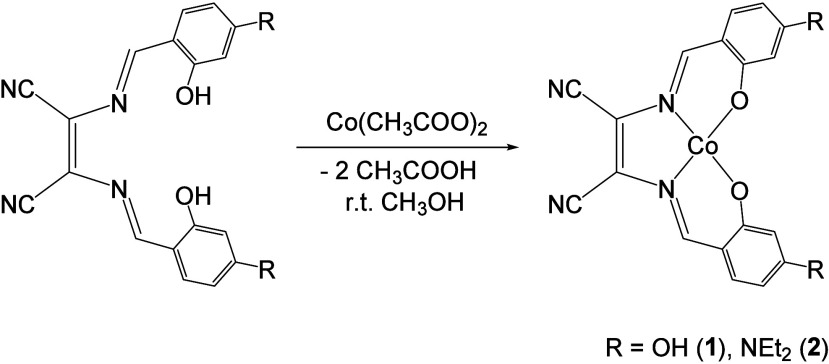
Structures of the Complexes under Investigation in This Study

Evidence for the formation of the complexes was achieved by MALDI-TOF MS spectrometry (Figures S1–S4). The mass spectrum of **1** shows a predominant peak at 427.99 *m*/*z* attributable to sodium adduct **1**Na^+^ on the basis of simulated mass and isotopic distribution patterns. A less intense peak was observed at 405.01 *m*/*z* corresponding to the molecular ion **1** ^+^ (Figures S1 and S2). Similarly in the mass spectrum of **2**, peaks at 515.16, 538.18, and 554.12 *m*/*z* were detected and are attributable to the molecular ion **2** ^**+**^, and to the sodium and potassium adducts **2**Na^+^, **2**K^+^, respectively (Figures S3 and S4). No peaks attributable to dimeric species were detected.

Because of the paramagnetism of the metal ion, the ^1^H NMR spectra of **1** and **2** only displayed strongly low-field-shifted resonances that were not useful for the characterization of the complexes. In the FT-IR spectra, the bands assigned to the stretching of the C=N bond (1632 cm^–1^) were shifted at lower wavenumbers compared to the same band in the ligands, as a consequence of complexation with cobalt­(II) (Figures S5 and S6).^23^

The UV spectra, at room temperature in DMSO, revealed three absorption bands in both the UV and visible regions (Figures S7 and S8); the bands in the UV region can be attributed to intraligand *n*→π* electronic transitions of the nonbonding electrons, whereas the bands in the visible region can be ascribed to intramolecular metal–ligand interactions *d*→π* within the complex.^24^ A further confirmation of the successful complexation can be obtained when comparing the UV–vis spectra of the free ligands with those of the complexes (Figures S9 and S10): shifts and changes of the absorption bands are evident in both cases. On excitation, both complexes exhibited luminescence with red emission at 645 nm. The fluorescence quantum yields (Φ_F_) of both complexes are similar to literature values reported for salen-based complexes^24^ and for the zinc analogues (see Table S1 in the Supporting Information).^18^

Despite several efforts, we did not succeed in obtaining single crystals suitable for X-ray diffraction analysis. To gain insight into the geometric and electronic structure, DFT calculations were undertaken. Cobalt­(II) complexes with the 3d^7^ electron configuration may have a low spin doublet (*S* = 1/2) or a high spin quartet ground state (*S* = 3/2). Gas phase calculation indicates that the ground state for complexes **1** and **2** is the doublet state, in agreement with experiment and DFT calculation carried out on related Co­(II)-salen complexes.^25,26^ The minimum energy structure of **1** is reported in Figure 1a. The cobalt ion adopts a square planar coordination geometry in which the computed bond distances are in good agreement with the experimental bond distances obtained from X-ray data of similar Co­(salen) complexes.^27^ The analysis of the electronic structure revealed that the SOMO is delocalized over the π-conjugated bonds of the ligand (Figure 1b) whereas the alfa-spin density (Figure 1c) is localized at the cobalt ion as well as at the imine nitrogen and phenoxo oxygen atoms, revealing a spin polarization of the electron density. An analogous coordination geometry and electronic structure were observed for complex **2**. In the gas phase, the quartet state lays only 2.1 and 2.2 kcal/mol above the doublet state for **1** and **2**, respectively. In these cases, a slight puckering of the N_2_O_2_ ligand backbone was observed that causes a slight elongation of both the Co–O and Co–N in-plane donor bond lengths.

**Figure 1:**
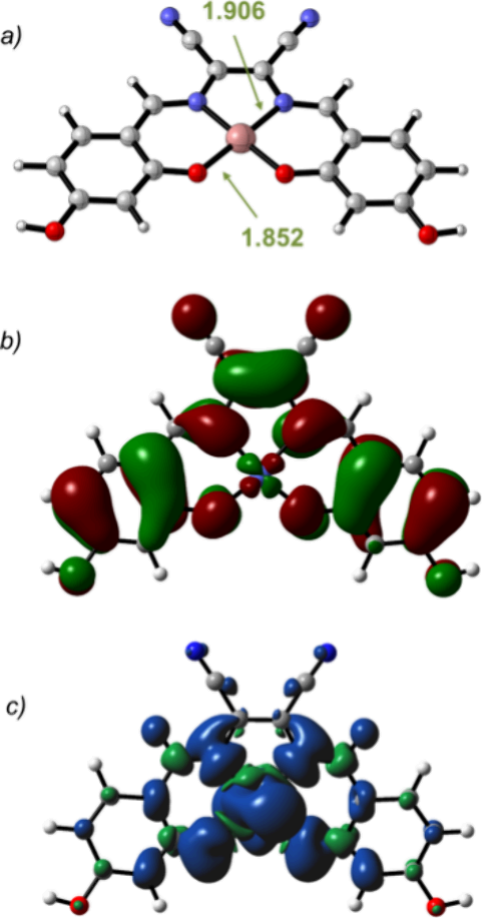
Minimum energy structures for complex **1** (a) and the same structure with the SOMO orbital (b) and total spin density (c). Distances are given in Å.

### HS^–^ Response of Complexes **1** and **2**

Interactions between complex **1** and complex **2** and HS^–^ were studied by UV–vis and fluorescence spectroscopy.

In the first instance, we tested the aptness of **1** or **2** to act as HS^–^ recognition elements by UV–vis spectroscopy. Figure 2 shows the spectral changes observed after the addition of HS^–^. When an excess of HS^–^ was added to a DMSO solution of **1** or **2**, a bathochromic shift in the absorption spectra was observed, indicating that new species are forming (Figure 2).

**Figure 2:**
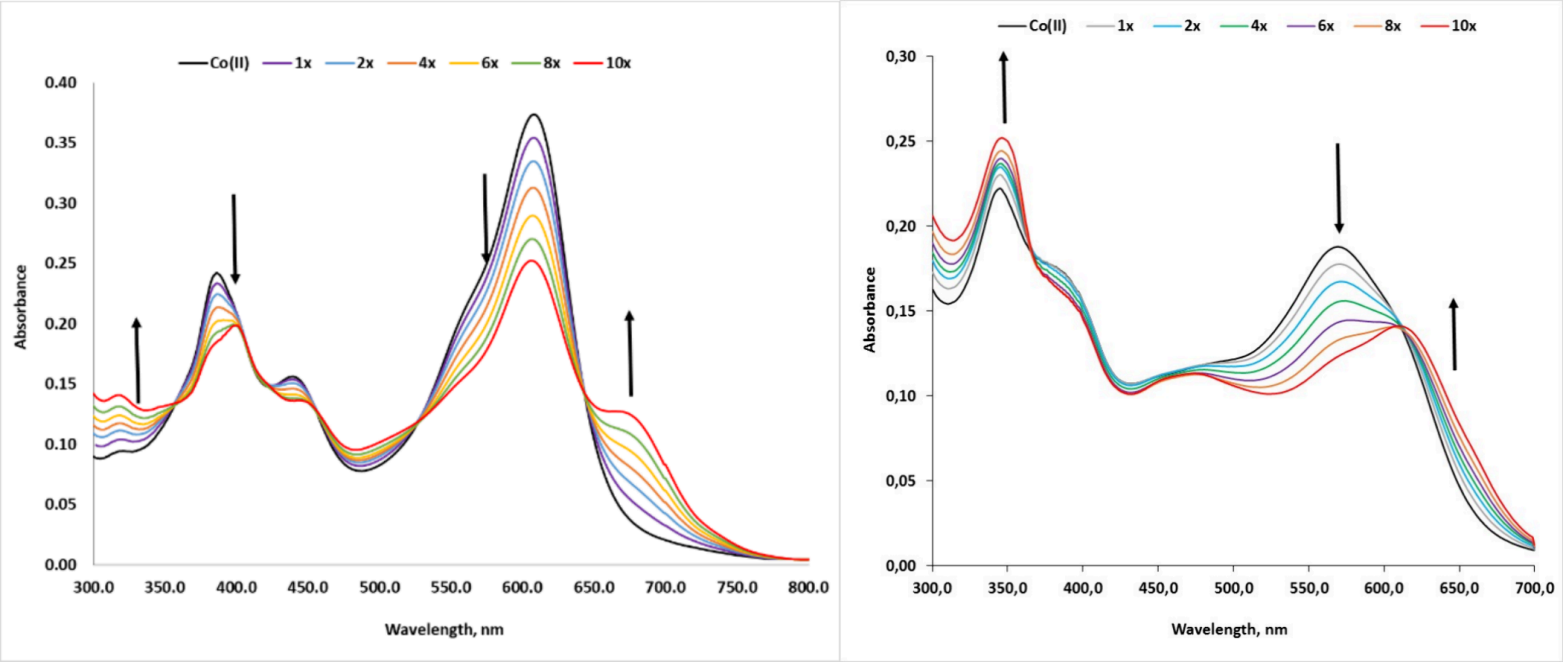
Absorption spectra of complexes **1** (left traces) and **2** (right traces) recorded in the presence of increasing equivalents of NaSH, ranging from a 1:1 to a 1:10 complex-to-NaSH molar ratio. Spectra were recorded in DMSO. [Complexes] = 10 μM for each spectrum. [NaSH] varied from 10 μM (1×) to 100 μM (10× relative to the complex concentration]).

When increasing amounts of HS^–^ were added and the electronic absorption spectra were monitored, a progressive change was observed, thus indicating a dose–response dependence for both the complexes. Titration of complexes **1** with NaSH in DMSO resulted in a significant bathochromic shift from 571 to 615 nm and well-anchored isosbestic points at 368 and 614 nm. Instead, titration of complexes **2** resulted in two significant bathochromic shifts from 611 to 678 nm and from 385 to 400 nm, and well-anchored isosbestic points at 357, 413, 423, 461, 527, and 645 nm (Figure 2). However, these new absorbances do not match the spectra of Co­(I) complexes generated from **1** or **2** by the addition of NaBH_4_ (Figures S11 and S12 in the SI), suggesting that HS^–^-mediated reduction of the metal or ligand is not occurring. The addition of NaBH_4_, a reductant stronger than HS^–^, failed to change the UV–vis spectrum of **1** and **2**: we only observed hypochromic effects. To test the reversibility of binding of HS^–^ to complexes **1** and **2**, the DMSO solutions of **1** or **2** treated with NaSH were exposed to atmospheric O_2_: the initial spectra were regenerated.

When the fluorescence intensities of complexes **1** and **2** were monitored in the presence of increasing amounts of HS^–^, again a dose–response dependence was found. More specifically, an enhancement of the initial fluorescence was observed for both the systems, and the fluorescence intensity increases together with the HS^–^ concentrations (Figure 3).

**Figure 3:**
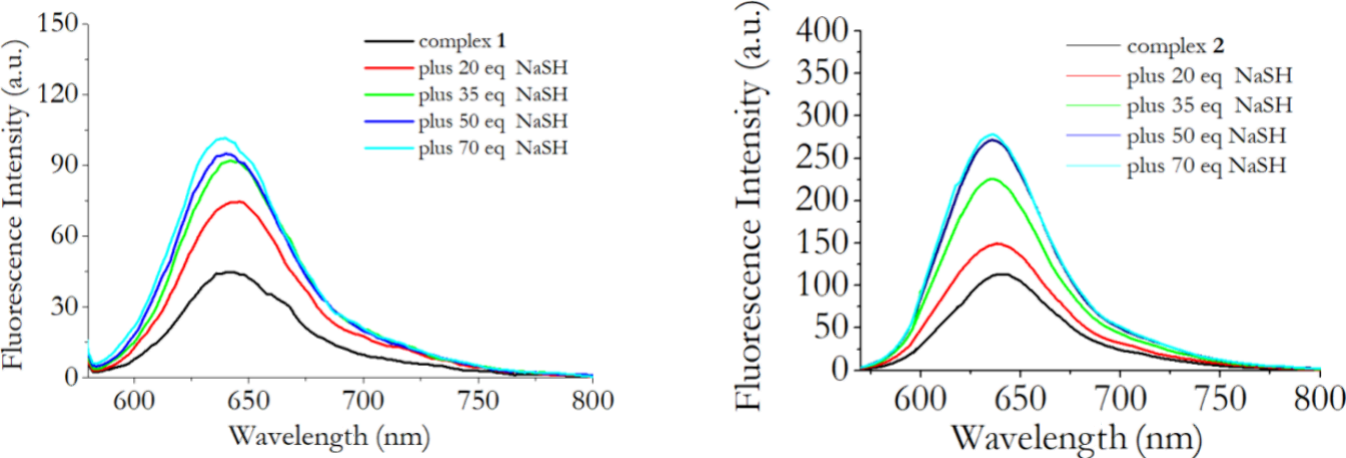
Emission spectra of complexes **1** and **2** before and after the addition of increasing amounts of NaSH. [Complexes] = 10 μM. All spectra were registered in DMSO with λ_exc_ = 570 nm for complex **1** (left traces); λ_exc_ = 610 nm for complex **2** (right traces).

When fitting the UV–vis (Figure 2) and fluorescence titration (Figure 3) data, the binding affinity constants for both complexes were determined using the Benesi–Hildebrand plot: log*K*_UV_ = (4.0 ± 0.1) and log*K*_FL_ = (3.7 ± 0.2) for complex **1** while log*K*_UV_ = (3.9 ± 0.1) and log*K*_FL_ = (3.7 ± 0.2) for complex **2** (see Supporting Material, Tables S2–S5). As experimental confirmation of the stoichiometry of HS^–^ binding to the title complexes, we performed a JOB PLOT experiment with complex **1**. The plot exhibits an inflection point at 0.5 (Figure S13), which points to a 1:1 stoichiometry for complex **1/HS** ^**–**^.

Next, to further test the systems, the fluorescence intensity of complex **1** was monitored as a function of time during a change from a HS^–^-rich to a O_2_-rich environment. Figure 4 shows a typical time trace of a solution containing 10 μM complex **1** when excited at the absorption maximum (λ = 570 nm).

**Figure 4:**
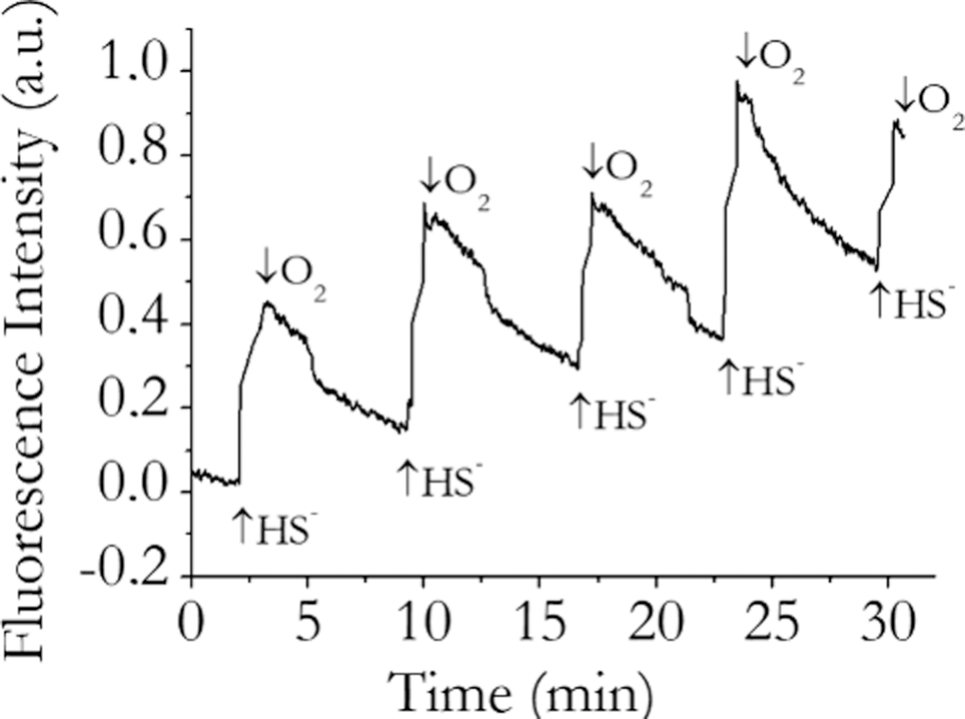
Room-temperature fluorescence intensity time trace observed at 645 nm (λ_ex_ 570 nm) of complex **1** upon addition of HS^–^ and of O_2_. [Complex **1**] = 10 μM; [NaSH] = 40 μM.

In this particular experiment, each cycle was started by adding HS^–^ to an end concentration of 40 μM (i.e., in excess over the complex concentration) and completed by passing oxygen through the solution. A fast increase in the fluorescence emission was clearly observed upon each HS^–^ addition. When bubbling through oxygen, the fluorescence intensity of the complexes diminished again; the cycle could be repeated many times. Most likely, exposure of the HS^–^ binding adducts of complexes **1** and **2** to atmospheric oxygen results in HS^–^ oxidation and regenerates the parent species, as already reported in the literature in the case of metallo-porphyrin systems.^21^ This finding showed that the HS^–^ sensing process is reversible, which is crucial for practical sensing applications.

In order to obtain a model for the coordination of HS^–^ to the cobalt complexes **1** and **2**, we resorted to DFT calculations. **1**/HS^–^ and **2**/HS^–^ were successfully located in both doublet and quartet spin states. For these adducts, the quartet states were significantly lower in energy with respect to the quartet states (Δ*E* = −21.5 and −11.2 kcal/mol for **1** and **2**, respectively), which is coherent with the previous observation that the coordination of an axial ligand in the Co­(II)-salen complex induces an increase of the energy gap between the two states.^25,26^ Both adducts display distorted square pyramidal coordination geometries with the cobalt cation lying in close proximity to the plane determined by the ONNO atoms of the ligands. The sulfur atom of the HS^–^ anions is in the apical position at the bond distances of 2.37 and 2.38 Å for **1** and **2**, respectively. The structure of **1**/HS^–^ in the quartet spin state is reported in Figure 5. The analysis of the electronic structure of **1**/HS^–^ showed that the SOMO is mainly localized over the sulfur atom (Figure 5b) whereas the alfa-spin density (Figure 5c) is delocalized over the sulfur, the cobalt, the imine nitrogen, and phenoxy oxygen atoms.

**Figure 5:**
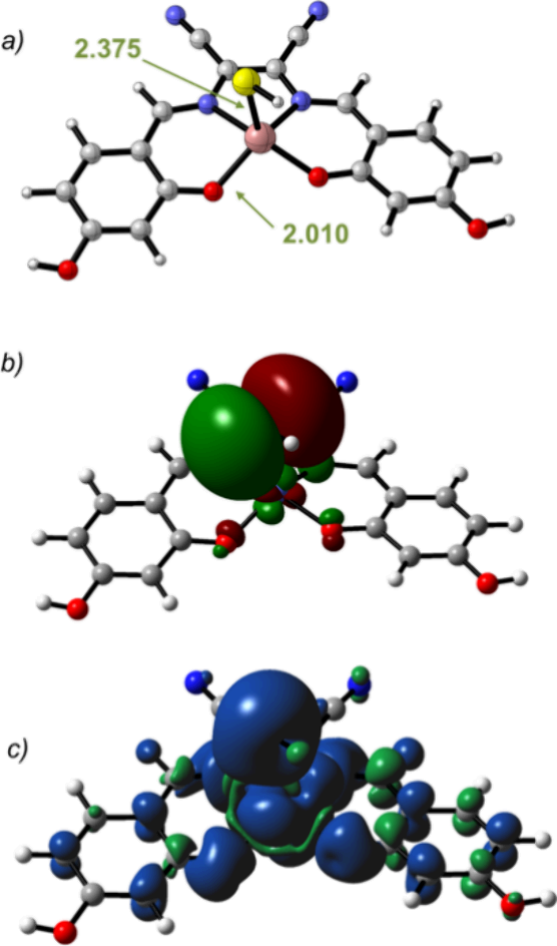
Minimum energy structures for complex **1/HS** ^**–**^ (a) and the same structure with the SOMO orbital (b) and the total spin density (c). Distances are given in Å.

### Electrochemical Evaluations

We investigated the effect of HS^–^ addition on the redox potential of the metal centers by performing cyclic voltammetry (CV) experiments on both Co­(II) complexes **1** and **2** (Figure 6). In DMSO with tetrabutylammonium trifluoromethanesulfonate of 0.1 M, the cyclic voltammograms in the −1.2 to +1.0 V potential range revealed two redox processes:

$${\left[L\mathrm{Co}\left(\mathrm{III}\right)\right]}^{+1}+e^{-}=\left[L\mathrm{Co}\left(\mathrm{II}\right)\right]$$ I

$$\left[L\mathrm{Co}\left(\mathrm{II}\right)\right]+e^{-}={\left[L\mathrm{Co}\left(I\right)\right]}^{-1}$$ II

**Figure 6:**
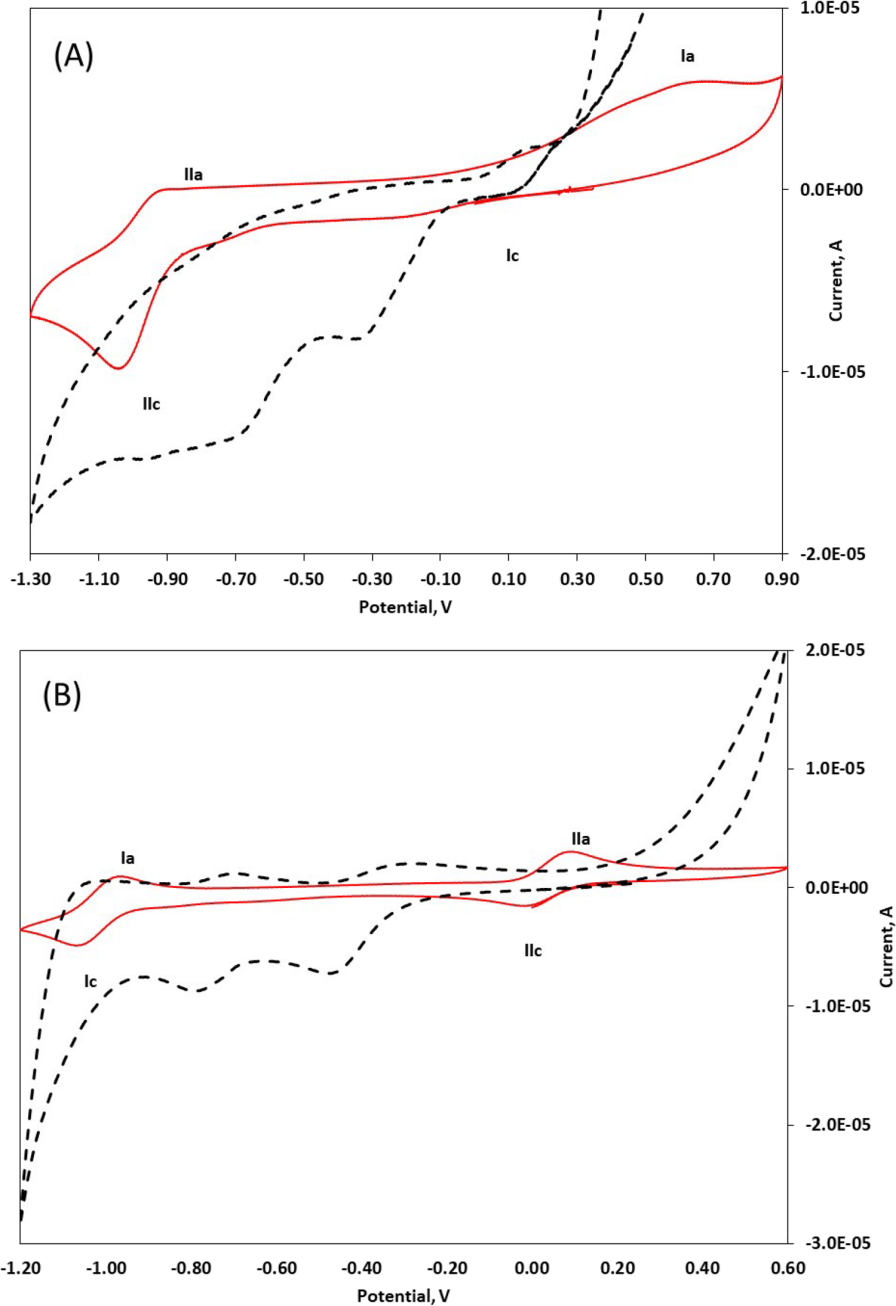
Comparison of the cyclic voltammograms of complex **1** (A) and **2** (B) in the absence (red lines) and presence (dotted lines) of HS^–^. The CVs were recorded in DMSO using a platinum working electrode for complex **1** and a glassy carbon working electrode for complex **2**. The experimental concentrations were [**1**] = 2.5 mM (red line) and [**1**] = 2.5 mM with [HS^–^] = 25 mM (dotted line) in panel (A); [**2**] = 1.9 mM (red line) and [**2**] = 1.9 mM with [HS^–^] = 19 mM (dotted line) in (B). All electrochemical measurements were referenced against an Ag/AgCl quasi-reference electrode. Supporting electrolyte: 0.10 M tetrabutylammonium trifluoromethanesulfonate. Scan rate = 50 mV/s.

No ligand reduction peaks are revealed in the potential window of the electrolytic medium. Furthermore, the subsequent addition of NaSH makes the window inaccessible above 1 V. The half-wave potential values, *E*_1/2_, of redox processes I and II of compounds **1** and **2** are reported in Table 1.

**Table 1:** Electrochemical Data Related to the Cyclic Voltammograms of Complexes **1** and **2** Reported in Figure 6

**half-reaction**	***E***_**1/2**_**(**V without HS	***E***_**1/2**_**(**V with HS	**delta (V)**	**redox process**
[*L*_1_Co(III)]^+^ + e^–^ = [*L*_1_Co(II)]	+0.24 quasi reversible wave	–0.08 quasi reversible wave	–0.32	Ia–Ic
[*L*_1_Co(II)]^+^ e^–^ = [*L*_1_Co(I)]^−1^	–1.00 quasi reversible wave	–0.48 quasi reversible wave	+0.52	IIa–IIc
[*L*_2_Co(III)]^+^ + e^–^ = [*L*_2_Co(II)]	+0.04 reversible wave	–0.38 quasi reversible wave	–0.42	Ia–Ic
[*L*_2_Co(II)]+ e^–^ = [*L*_2_Co(I)]^−1^	–1.01 reversible wave	–0.73 quasi reversible wave	+0.28	IIa–IIc

For the compound [*L*_1_Co­(II)], only the reduction signal IIc is clearly evident, while the oxidation signals Ia and IIa and the reduction signal IIc are less intense (Figure 6A). Both processes are quasi reversible. A large peak-to-peak separation was observed for redox process I. Instead, for the compound [*L*_2_Co­(II)], two reduction signals Ic and IIc and two oxidation signals Ia and IIa can be observed (Figure 6B), and both processes are reversible. We then investigated the redox behavior of complexes **1** and **2** in HS^–^ large excess (10×). The addition of this excess of HS^–^ (i) results in an enhancement of the peak currents, (ii) causes any shift in the peak potentials, or (iii) results in the appearance of any additional peaks in the cyclic voltammograms of complex **1**. For both complexes, it is evident that both processes are quasi reversible, all the oxidation processes are less evident, and significant anodic and cathodic shifts were observed for processes I and II, respectively. These results suggest that HS^–^ coordination provides additional stability to metal centers in higher oxidation states.^28^ Using the potential of the ligand as a reference, it is fair to assume that the addition of the HS^–^ can affect the electrochemical properties of the cobalt­(II) metal center. The potential for the Co^III/II^ and Co^II/I^ redox couples is shifted toward zero (vs SSE) in both complexes and suggests that the addition of HS^–^ makes the complex more accessible to oxidation and reduction processes. From these findings, we have clearly shown that CV can be used to qualitatively assess that HS^–^ anion coordinates to the Co­(II) metal center in the cobalt DAMN complex.^29^ These Co­(II)­L-HS species are very sensitive to oxygen, and through the CV experiments, we found that if the oxygen was added for 15 min, then the oxidation of HS^–^ proceeded: very similar voltammograms of the redox processes I and II are recorded (not shown).

When performing the CV experiments with complexes **1** and **2** in the presence of an excess of NaBH_4_, the redox signals associated with the Co­(II) oxidation state clearly disappear (see Figures S14 and S15).

As an independent confirmation that complex **1** is binding the HS^–^ anion, we resorted to ^77^Selenium NMR spectroscopy, considering that selenium is a congener of sulfur and these two elements could feature a similar chemical reactivity. Due to the scarce solubility of Na_2_Se in most organic solvents, we tracked the reaction between complex **1** and benzenselenol (C_6_H_5_SeH). The ^77^Se NMR spectrum of C_6_H_5_SeH, reported in Figure 7, displays a unique resonance at 449.6 ppm. Upon the addition of 1 equiv of complex **1**, the signal was shifted to a lower chemical shift by 1.4 ppm. To rule out the possibility that this shift is due to changes in the magnetic susceptibility of the environment, the concentration of C_6_H_5_SeH was varied while the concentration of complex **1** was kept constant. As shown in Figure 7, a shift in the signal was observed that is consistent with the presence of a coordination equilibrium between complex **1** and C_6_H_5_SeH occurring in a fast exchange regime.

**Figure 7:**
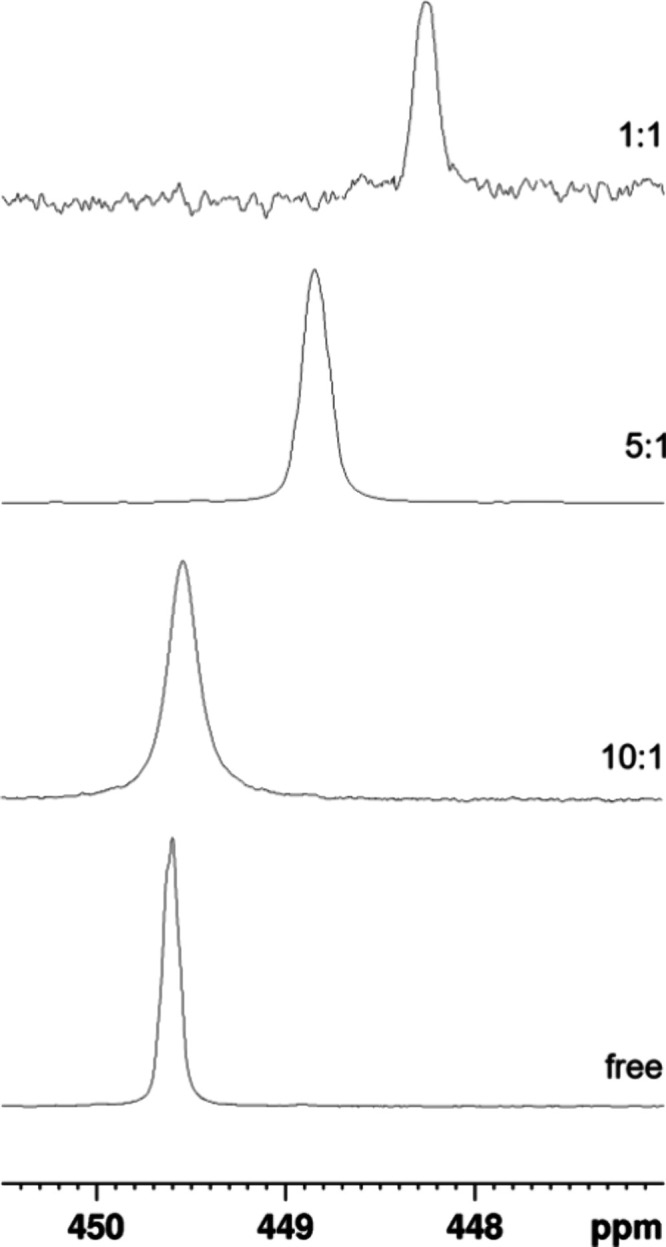
Stacking plot of partial ^77^Se NMR spectra of C_6_H_5_SeH/**1** at different molar ratios ([**1**] = 20 mM, DMSO-d_6_, rt, 114.4 MHz).

### Selectivity of Complexes **1** and **2** against HS^–^

To assess the selectivity of the title complexes toward HS^–^, we monitored the fluorescence of the systems in the presence of different anions. Figure 8 displays the obtained results.

**Figure 8:**
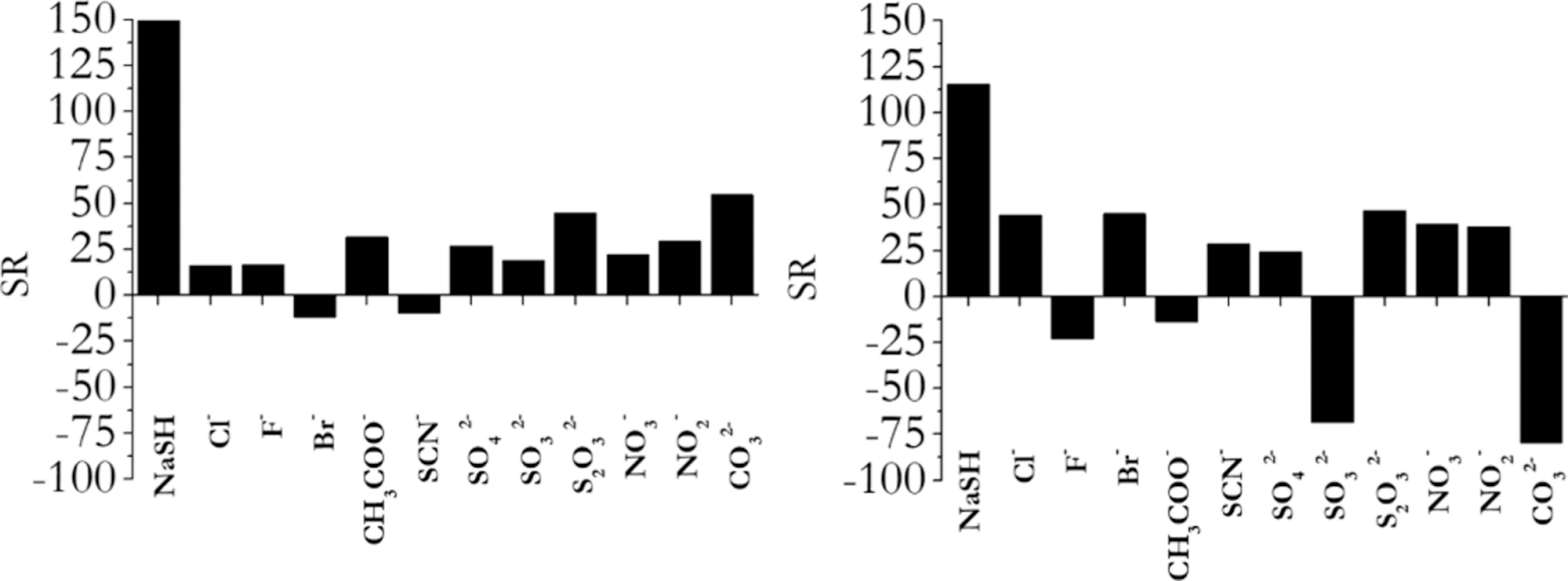
Relative fluorescence emission at 635 nm (λ_ex_ = 570 nm) of complex **1** (histogram on the left) and at 640 nm (λ_ex_ = 610 nm) of complex **2** (histogram on the right) in the presence of 1 equiv of HS^–^ and of 10 mM of other analytes in aqueous solution [complexes] = 25 μM. SR (switching ratio) is given by *F*_max_–*F*_min_/*F*_max_.

The above results point to selectivity in the response to HS^–^ of both complexes under investigation in the experimental conditions tested: a fluorescence enhancement lower than that observed with HS^–^ or, in some cases, a fluorescence quenching of the initial fluorescence intensity was observed in the presence of other anions of biological relevance tested.

In a different experiment, we monitored the fluorescence responses of complexes **1** and **2** to HS^–^ in the presence of the same competitors tested in the experiment in Figure 8. Figure 9 displays the obtained results. Indications of a selective response could be drawn also in this case: an evident fluorescence enhancement was observed when an excess of NaSH was added in the presence of the several competitor species.

**Figure 9:**
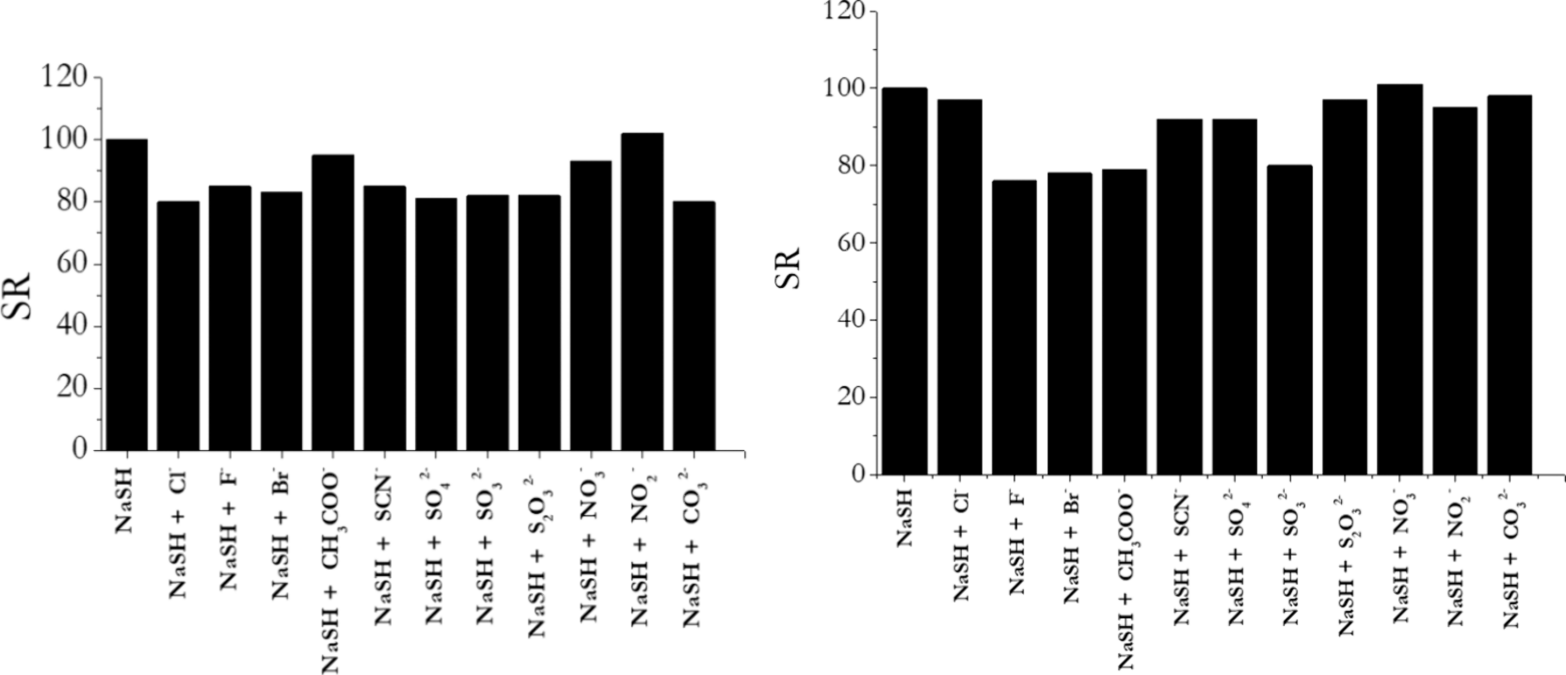
Anti-interference study for complex **1** (histogram on the left) and complex **2** (histogram on the right) in the presence of 400 μM NaSH and other analytes (10 mM) [complexes] = 25 μM. SR (switching ratio) is given by *F*_max_–*F*_min_/*F*_max_.

Similar indications pointing to a selective response of complexes **1** and **2** to HS^–^ were obtained when the colorimetric responses to HS^–^ were monitored (Figure 10). As clearly visible from Figure 9, specific color variations, different than those observed for other anions, were observed for both complexes in the presence of HS^–^.

**Figure 10:**
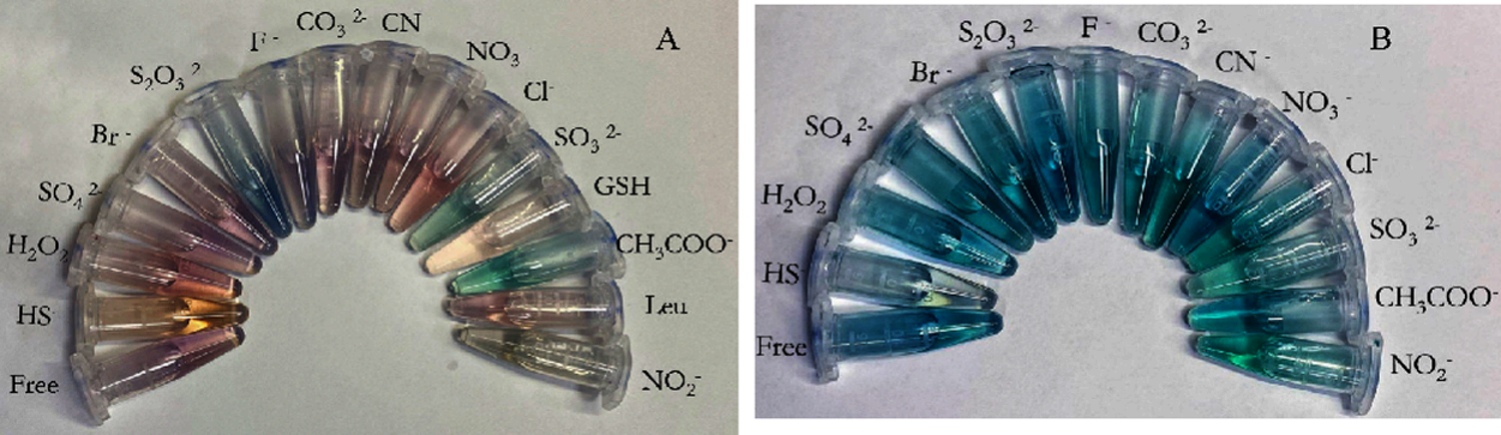
Colorimetric response for complex **1** (reported in part A) and complex **2** (reported in part B) in the presence of the anions indicated in the figures [complexes] = 25 μM; [NaSH] = 400 μM and [anions] = 10 mM.

For a good molecular probe, further than selectivity, sensitivity is also an essential feature: the limit of detection (LOD) was found in the micromolar range for both complexes **1** and **2** (Figure S16 and Table S6).

### Biological Assays in Living Cells

With these results in hands, encouraged by the reversible fluorescence response to HS^–^ observed in the cuvette experiments (see Figure 4), and encouraged by the finding that complex **2** resulted more fluorescent than complex **1** in the experimental conditions tested (see Figure 3 and Table S1), we explored its potential to visualize exogenous H_2_S in HepG2 cells in a reversible manner. As mentioned above, to our knowledge, reversible systems that function in complex environments, such as live cells or tissues, remain a challenging and unmet task.^7^

Before starting the imaging experiments with complex **2**, we assessed the cytotoxicity of our probe *via* an MTT experiment. The MTT assay in HepG2 cells showed that complex **2** was slightly toxic under the experimental conditions tested (Figure S17).

In a second instance, to explore whether complex **2** could permeate the cells, we incubated the cells for 60 min with our probe (90 μM). Figure 11A shows the cells after treatment with the sensing complex, and as evident, cells displayed red fluorescence, thus indicating that the probe entered the cells. To investigate the ability of complex **2** to visualize exogenous H_2_S in living cells, we compared the fluorescence of the cells incubated with complex **2** with that of the cells incubated with complex **2** and then treated with 260 μM NaSH (comparable with physiological concentrations)^30–32^ to allow the intracellular formation of HS^–^. A further fluorescence enhancement was observed (Figure 11B), demonstrating the capability of the probe to detect HS^–^ inside cells directly. We then incubated the cells with oxygen-saturated DMEM and observed an evident quenching of the fluorescence (Figure 11C). When incubating for the second time the cells with 260 μM NaSH, a light fluorescence enhancement was again visible (Figure 11D) as was the fluorescence quenching when incubating the cells with oxygen-saturated DMEM (Figure 11E).

**Figure 11:**
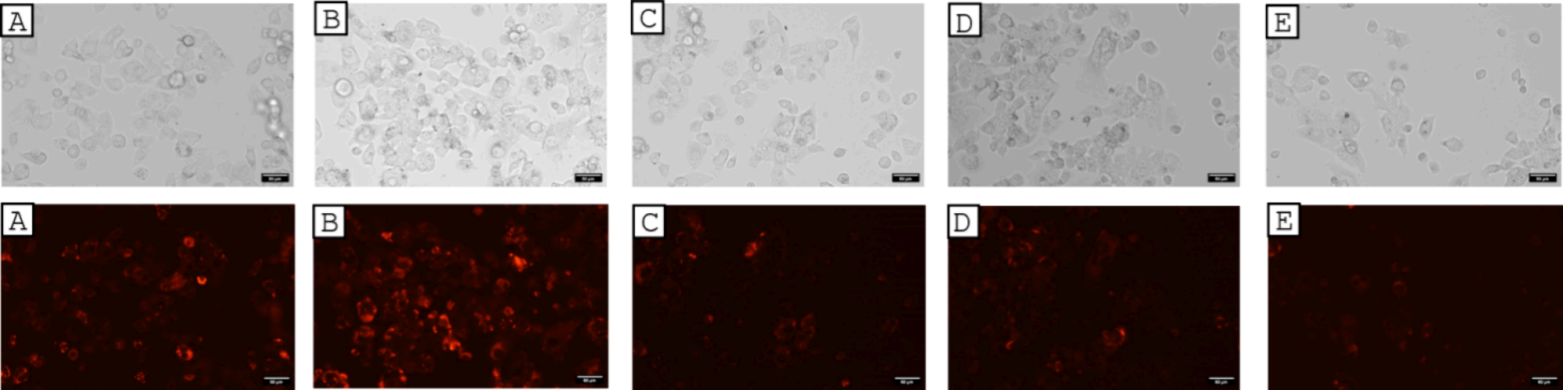
Fluorescence microscopy images of HepG2 cells after 1 h treatment with 90 μM complex **2** (indicated as A), 90 μM complex + 260 μM NaSH (exogenous HS^–^) (indicated as B), 90 μM complex **2** + 260 μM NaSH (exogenous HS^–^) after 30 min incubation with oxygen-saturated DMEM (indicated as C), 90 μM complex after 30 min of incubation with 260 μM NaSH (exogenous HS^–^) (indicated as D), and 90 μM complex after 30 min incubation with oxygen-saturated DMEM (indicated as E). Magnification 20×.

These experiments indicate that complex **2** also works as a reversible indicator of H_2_S inside the cells. As a control experiment, we also checked that nontreated HepG2 cells were not fluorescent (Figure S18).

To gain quantitative evidence of the fluorescence switchings observed in Figure 12, we investigated the fluorescence intensity and area through the ImageJ software (Figure 12). The results evidence a raise in the initial fluorescence intensity of cells incubated with complex **2** after the addition of exogenous H_2_S and a quenching after the treatment with oxygen-saturated HBSS.

**Figure 12:**
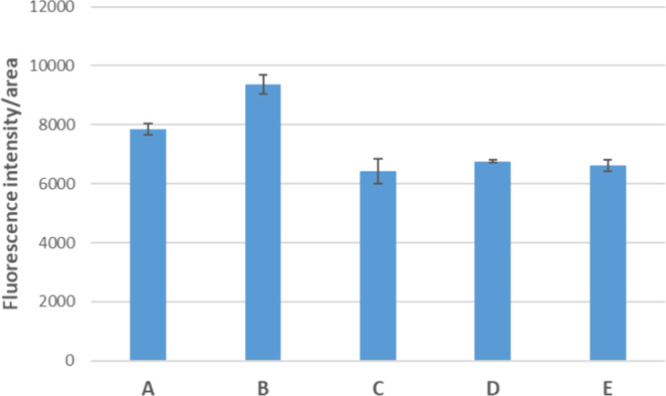
Quantification of fluorescence intensity/area in the presence of complex **2** (indicated as A) compared to cells treated with complex **2** and exogenous H_2_S (indicated as B), cells treated with complex **2** and HBSS oxygenated (indicated as C), cells treated with complex **2** and exogenous H_2_S (indicated as D), and cells treated with complex **2** and HBSS oxygenated (indicated as E).

In a different experiment, always looking at fluorescence of HepG2 cells and aiming at a more quantitative response, we studied the probe potential with a highly sensible instrument such as SpectraMaxMiniMulti-Mode Microplate Reader (Molecular Devices) by dissolving complex **2** in MEM buffer, in concentrations close to biological conditions. Figure 13 displays the obtained results.

**Figure 13:**
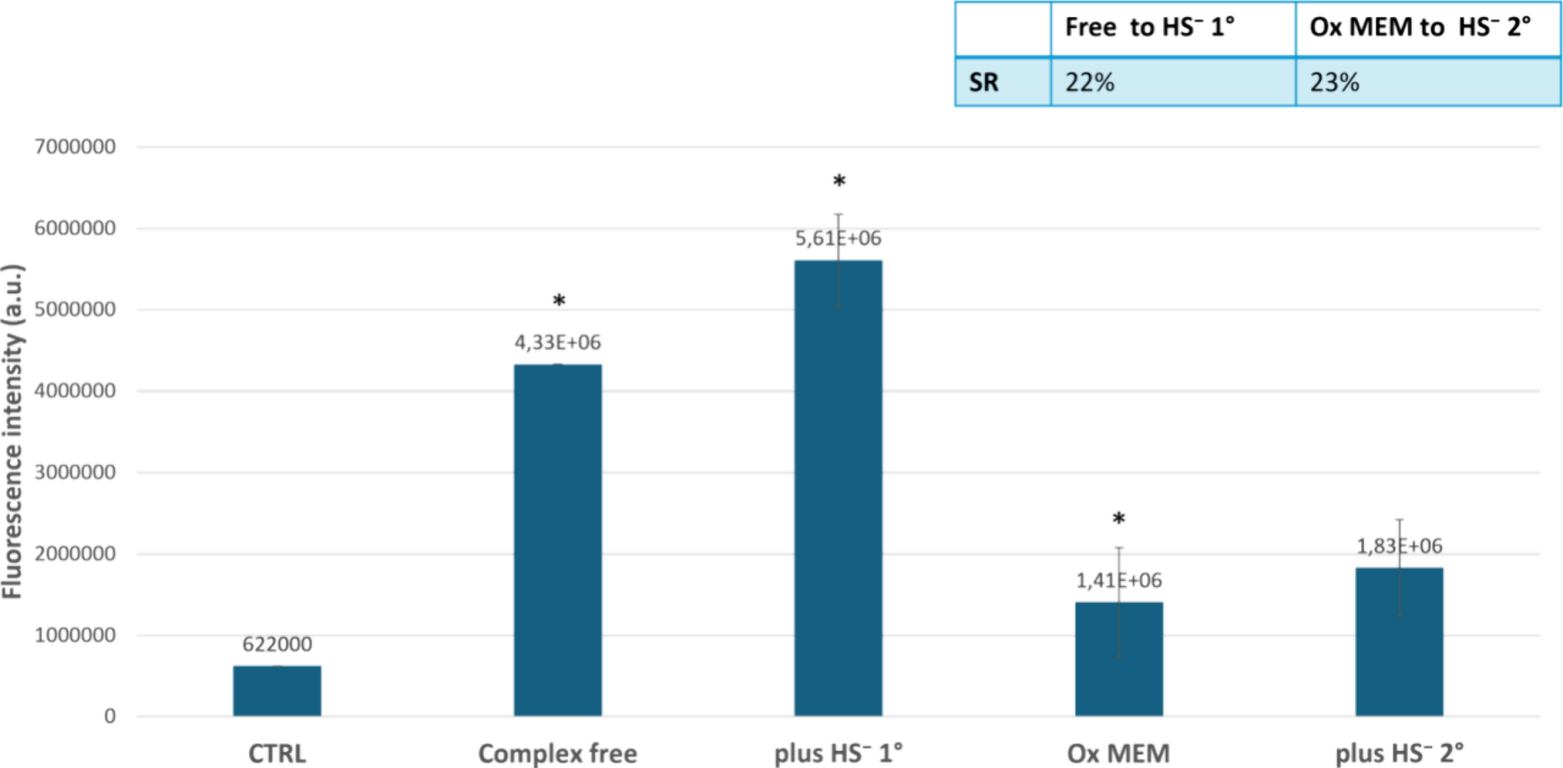
Fluorescence intensity in the presence of nontreated cells (indicated as CTRL), cells treated with complex **2** (indicated as Complex free), cells treated with complex **2** and 260 μM of exogenous H_2_S (indicated as plus HS^–^ 1°), cells treated with complex **2** and HBSS oxygenated (indicated as Ox MEM), and cells treated with complex **2** and 260 μM of exogenous H_2_S (indicated as plus HS^–^ 2°).

As already visible from the imaging experiments, fluorescence intensity of the cells experiences an increase in the presence of complex **2** after addition of exogenous H_2_S and also after the fluorescence quenching promoted by the oxygenated buffer (which is quite evident); when adding again an amount of HS^–^, in the second cycle of detection, the fluorescence enhancement of cells after addition of HS^–^ is still visible (Figure 13). This finding showed that the H_2_S binding process is reversible also in living cells, which is crucial for practical sensing applications; a limitation is that the maximum and minimum fluorescence levels decrease with the number of cycles in a single experiment: for practical applications, the aim is to enhance these fluorescence differences.

### Smart Application: Detecting H_2_S in Common Drinks

A further application for the sensing complexes proposed here was to assay their ability to result in a fluorescence signal when the HS^–^ is dissolved in either tap water or beer or commercial tea. For both complexes **1** and **2**, progressive fluorescence enhancements were observed also in these experimental conditions. Figures S19 and S20 display the related fluorescence intensity traces.

## Conclusions

New fluorescent salen-based cobalt complexes have been synthesized and characterized, and their reactivity with HS^–^ was explored *via* different spectroscopic techniques. We provide evidence that HS^–^ binds the Co center of both of the complexes under investigation. The different substituents on the phenolate moieties do not significantly affect the reactivity with the target analyte of the two complexes under investigation; the most relevant difference is the fluorescent intensity of the starting complex, which is higher for complex **2**, and most likely this is due to the diethyl-amine groups on the ligand framework. A reversible binding of HS^–^ to the cobalt centers was found when passing from a HS^–^-rich to an oxygen-saturated environment, which in turn results in the modulation of the fluorescence intensity: a fast fluorescence enhancement was observed upon each HS^–^ addition, whereas the fluorescence intensity diminished again when bubbling through oxygen. Proof-of-principle results showing that modulation of the fluorescence intensity in response to the HS^–^ can also be achieved in a cell culture are also presented.

To the best of our knowledge, this is the first study reporting Co­(salen-type) complexes functioning as fluorescent HS^–^ sensors *via* a "coordination-based" approach. The successful increase and quenching of the fluorescent response of the complexes when passing from an HS^–^-rich to an oxygen-rich environment or, in other words, cyclability of the system, is also a big advantage for practical measurements. We believe that providing a proof of principle that cyclability of the systems under investigation is still valid in living cells is the most promising result of the present work and was also reported here for the first time.

We foresee that these findings may open the door to new applications beyond H_2_S sensing.

## Experimental Section

### Materials

All chemicals used for the synthetic work were obtained from Sigma-Aldrich or Strem Chemicals and were of reagent grade. They were used without further purification. Synthesis of proligands, 3-bis­[[(2,4-dihydroxy-phenyl)­(methylene)]­amino]-2-butenedinitrile (*L*_1_-H) and 2,3-bis­[[(2-hydroxy-4-(diethylamino)­phenyl)­(methylene)]­amino]-2-butenedinitrile (*L*_2_-H), was performed by following literature procedures.^18,33–36^

### General

HR MALDI mass spectra were recorded using a Bruker solariX XR Fourier transform ion cyclotron resonance (FT-ICR) mass spectrometer (Bruker Daltonics GmbH, Bremen, Germany) equipped with a 7 T refrigerated actively shielded superconducting magnet (Bruker BioSpin, Wissembourg, France). The samples were ionized in positive or negative ion mode by using the MALDI ion source. The mass range was set to *m*/*z* 150–2000. The laser power was 15%, and 15 laser shots were used for each scan. Mass spectra were calibrated externally using a mix of peptide clusters in the MALDI ionization positive ion mode. A linear calibration was applied.

CV experiments were performed using an Autolab PGSTAT502F Electrochemical Analyzer (Metrohm). All electrochemical studies were referenced with respect to a Ag/AgCl reference electrode. All potential values were calibrated against the saturated silver electrode (SSE) by measuring the oxidation of ferrocene as an internal reference (*E* ^o^(Fc^+^/Fc) = 0.424 V vs SSE)­1.^37^ Platinum and glassy carbon disk (diameter: 1 mm) working electrodes were used for electrochemical studies for complex I and complex II, respectively. They were polished with 0.05 μM alumina on a felt pad, sonicated in absolute ethanol, and a deionized water mixed solution (1:1 v/v) for 5 min. Then, it was rinsed with ethanol and deionized water before each measurement. Solutions with an excess of HS^–^ were prepared by adding solid NaSH, weighed with the precision of 0.01 mg. All of the electrochemical samples were purged with Ar gas for 10 min and were measured under an Ar atmosphere. Basically, CV was performed on dimethylsulfoxide (DMSO) solution samples with 0.10 M of tetrabutylammonium trifluoromethanesulfonate as supporting electrolyte.

#### Synthesis and Characterization of Complex **1**

To a stirred solution containing the ligand precursor (0.348 g, 1.0 mmol) in CH_3_OH (50.0 mL) was added dropwise a solution of (CH_3_COO^–^)_2_Co (0.274 g, 1.1 mmol) in CH_3_OH (5.0 mL), and then the solution was stirred overnight at room temperature in a N_2_ atmosphere. All volatiles were then removed in vacuo. Recrystallization of the crude product from 1:1 ethanol/pentane afforded **1** as a dark-red solid. The formation of the desired species was confirmed by MALDI. MS (MALDI FT-ICR, MeOH): *m*/*z* (%) calculated: 405.002 experimental: 405.006 [complex **1**]^+^. UV–visible bands (DMSO): 345, 384, and 570 nm. Anal. calcd for complex **1** C_18_H_10_CoN_4_O_4_: C, 53.35 H, 2.49; N, 13.83. Found: C, 53.40; H, 2.39; N, 13.79.

#### Synthesis and Characterization of Complex **2**

To a stirred solution containing the ligand precursor 3-bis­[[(2,4-dihydroxy-phenyl)­(methylene)]­amino]-2-butenedinitrile (*L*_1_-H) (0.204 g, 0.50 mmol) in CH_3_OH (25.0 mL) was added dropwise a solution of (CH_3_COO^–^)_2_Co (0.136 g, 0.55 mmol) in CH_3_OH (5.0 mL), and then the solution was stirred overnight at room temperature in N_2_ atmosphere. All volatiles were then removed in vacuo. Recrystallization of the crude product from 1:1 ethanol/pentane afforded **2** as a dark-green solid. The formation of the desired species was confirmed by MALDI. MS (MALDI FT-ICR, MeOH): *m*/*z* (%) calculated: 515.160 experimental: 515.160 [complex **2**]^+^. UV–visible bands (DMSO): 384, 439, and 608 nm. Anal. calcd for complex **2** C_26_H_28_CoN_6_O_2_: C, 60.58 H, 5.48; N, 16.30. Found: C, 60.63; H, 5.39; N, 16.35.

##### Absorbance and Fluorescence Measurements

Absorption spectra were recorded on a Cary 50 spectrophotometer, using a 1 cm quartz cuvette (Hellma Benelux BV, Rijswijk, Netherlands) and a slit width equivalent to a bandwidth of 5 nm. Fluorescence spectra were measured on a Cary Eclipse Spectrophotometer in a 10 × 10 mm^2^ airtight quartz fluorescence cuvette (Hellma Benelux BV, Rijswijk, Netherlands) with an emission bandpass of 10 nm and an excitation bandpass of 5 nm. For the fluorescence experiments, PMT voltage was set to 500 V, unless differently stated in the caption of the figure. Both absorption and fluorescence measurements were performed either in DMSO or in Milli-Q water solutions at 25 °C. Fluorescence emission spectra were registered by exciting the samples at a specific wavelength (as stated in the figure captions).

Fluorescence quantum yield (Φ_F_) values were measured in optically diluted solutions using as standards the commercial dyes Cy3 NHS (Φ_F_ = 0.15 in Milli-Q water), according to the equation^38^

$${\Phi_{F}}^{s}={\Phi_{F}}^{r}\left(I_{s}/I_{r}\right)\left(A_{r}/A_{s}\right){\left(\eta_{s}/\eta_{r}\right)}^{2}$$

where indexes s and r denote the sample and reference, respectively. *I* stands for the integrated emission intensity, *A* is the absorbance at the excitation wavelength, and η is the refractive index of the solvent. The optical density of the complexes and standards was kept below 0.1. The uncertainty in the determination of Φ_F_ is ± 15%.

The fluorescence intensity kinetic experiments were performed as follows: the cuvette was filled with the sample solution in DMSO, and the experiment was started. Then, microliter amounts of HS^–^ solution (to the end concentrations specified in the figure captions) were injected *via* a gastight syringe and the fluorescence intensity changes accordingly, until a constant value. Subsequently, a gentle flow of O_2_ was directed in the cuvette until the fluorescence intensity reaches the initial value. The cycle was repeated several times.

##### Computational Details

Molecular structures and electronic energies were calculated using the Gaussian 09 ^39^ package at the B3LYP level of theory. The basis set consisted of the Los Alamos basis sets and corresponding effective core potentials of Hay and Wadt^40,41^ (LANL2DZ) for Co and of the 6-31G­(d) basis sets for all other atoms. A tight gradient convergence criterion with an ultrafine integration grid was specified in these calculations. No constraints were imposed during the optimizations. Stationary point geometries were characterized as the local minimum on the potential energy surfaces. The absence of imaginary frequency demonstrated that structures were true minima at their respective levels of theory.

The reported Gibbs free energies have been obtained adding thermal corrections in the gas phase to electronic energy in solvent (PCM model)^42^ computed via single-point calculation in DMSO at the B3LYP level with the def2TZVPP basis set for cobalt and the 6-311+G­(2d,p) basis set for all other atoms. Cartesian coordinates of all DFT-optimized structures are available upon request.

##### Fluorescence in MEM

Before fluorescence imaging experiments, we studied probe potential in solvents and concentrations close to biological conditions as MEM with a highly sensible instrument as SpectraMax Mini Multi-Mode Microplate Reader (Molecular Devices). We seeded HepG2 cells at 15,000 cells/well. The day after, we prepared a 1 mg/mL probe solution dissolving complex **2** in DMSO, and then we diluted the stock solution in MEM to have increasing concentrations of the probe. We added 100 μL for each well in a 96-multiwell plate for each sample. Only for probe-HS^–^ samples did we add NaSH dissolved in H_2_O, and after 30 min, we rinsed and added the oxygen-saturated MEM. We analyzed fluorescence intensities with the SpectraMax Mini Multi-Mode Microplate Reader (Molecular Device) for each sample.

##### Cell Culture

HepG2 cells (human hepatocellular liver carcinoma cell line) were grown in minimum essential medium (MEM) supplemented with 10% fetal bovine serum (FBS), 2 mM glutamine, 1 mM nonessential amino acids, and 1% antibiotics (penicillin/streptomycin, 100 U/mL). Cells were maintained in a humidified incubator at 37 °C, in 5% CO_2_/95% air. 1.5 × 10^5^ cells/well were seeded on 12-well multiwell plates 1 day before imaging.

##### Fluorescence Imaging

To verify the loading of the probe and capability in H_2_S detection, HepG2 cells were incubated with 90 μM complex **2** diluted in cell culture medium for 1 h at 37 °C. After incubation, cells were rinsed to remove an excess of complex. Probe-loaded cells were observed by an automated inverted fluorescence microscope (Olympus IX83) at 545–557 nm excitation wavelength using a 10× objective and a 20× objective. Only probe-loaded cells were further treated with exogenous NaSH (260 μM in MEM) for 30 min and then observed with the microscope to test the capability of the complex to monitor the intracellular increase of H_2_S.

To have quantitative evidence of the fluorescence enhancement, we analyzed the fluorescence intensity/area through ImageJ software. We set five fixed areas and measured the fluorescence intensity for each sample.

##### Calculation of the LOD

In agreement with the IUPAC recommendation,^43,44^ the LOD for HS^–^ was calculated from the spectrofluorometric titration data, for both complexes **1** and **2**, using the following equation:

LOD=3s/K

where *s* and *K* represent, respectively, the standard deviation of the blank and the absolute value of the slope of the calibration line (Figure S16).

## Supplementary Material



## Notes

MALDI, IR, and absorption and fluorescence spectra; cytotoxicity evaluation; fluorescence microscopy images of HepG2 cells; and UV–vis and fluorescence fitting data (PDF)
