# Supplementary material for: Diaminomaleonitrile (DAMN)-Based Salen-Type Cobalt Complexes as Cyclable Sensors for H2S/HS–: Biological Applications
Source: Inorg Chem. 2025 May 13;64(20):10260–71. doi: 10.1021/acs.inorgchem.5c01186 (PMC12117558; doi:10.1021/acs.inorgchem.5c01186)
Supplement: Supplementary file 1 [file ic5c01186_si_001.pdf]

Diaminomaleonitrile (DAMN)-based salen-type cobalt complexes as cyclable sensors for H_2S/HS^-: biological applications.

Alessio Trerotola[a], Viktoriia Vykhovanets[a], Tonino Caruso[a], Daniela Guarnieri[a], Marina Lamberti[a], Stefano Milione[a]*, Maria Strianese.[a]*

[a]Dipartimento di Chimica e Biologia "Adolfo Zambelli" and INSTM Research Unit, Università degli Studi di Salerno, Via Giovanni Paolo II, 132, 84084 Fisciano (SA) Italy.

Supporting information

*Corresponding authors. E-mail: mstriane@unisa.it; smilione@unisa.it

Contents:

Figure S1	MALDI spectrum of complex **1**	S3
Figure S2	Enlargement of the MALDI spectrum of complex **1**	S4
Figure S3	MALDI spectrum of complex **2**	S5
Figure S4	Enlargement of the MALDI spectrum of complex **2**	S6
Figure S5	IR spectrum of L_1-H and complex **1**	S7
Figure S6	IR spectrum of L_2-H and complex **2**	S8
Figure S7	UV-vis spectrum of complex **1**	S9
Figure S8	UV-vis spectrum of complex **2**	S10
Figure S9	UV-vis spectra of L_1-H and complex **1**	S11
Figure S10	UV-vis spectra of L_2-H and complex **2**	S12
Figure S11	UV-vis spectrum of complex **1** in the presence of $NaBH_4$	S13
Figure S12	UV-vis spectrum of complex **2** in the presence of $NaBH_4$	S14
Figure S13	Job's plot for complex **1**/HS^-	S15
Figure S14	Cyclic voltammogram of complex **1** in the absence and presence of $HS^−$ or BH_4^-	S16
Figure S15	Cyclic voltammogram of complex **2** in the absence and presence of $HS^−$ or BH_4^-	S17
Figure S16	Linear best fits of calibration line for the calculation of LOD for HS^-	S18
Figure S17	Cytotoxicity evaluation of complex **2** in HepG2 cell line by MTT assay	S19
Figure S18	Fluorescence microscopy images of HepG2 cells	S20
Figure S19	Emission spectra of complex **1** in common drinks	S21
Figure S20	Emission spectra of complex **2** in common drinks	S22
Table S1	Photophysical features of the title complexes	S23
Table S2	UV-vis fitting data for the determination of the binding constant between complex **1** and $HS^−$ *via* a Benesi-Hildebrand plot	S24
Table S3	Fluorescence fitting data for the determination of the binding constant between complex **1** and $HS^−$ *via* a Benesi-Hildebrand plot	S25
Table S4	UV-vis fitting data for the determination of the binding constant between complex **2** and $HS^−$ *via* a Benesi-Hildebrand plot	S26
Table S5	Fluorescence fitting data for the determination of the binding constant between complex **2** and $HS^−$ *via* a Benesi-Hildebrand plot	S27
Table S6	The standard deviation of the blank, the absolute value of the slope of the calibration line and LOD values	S28

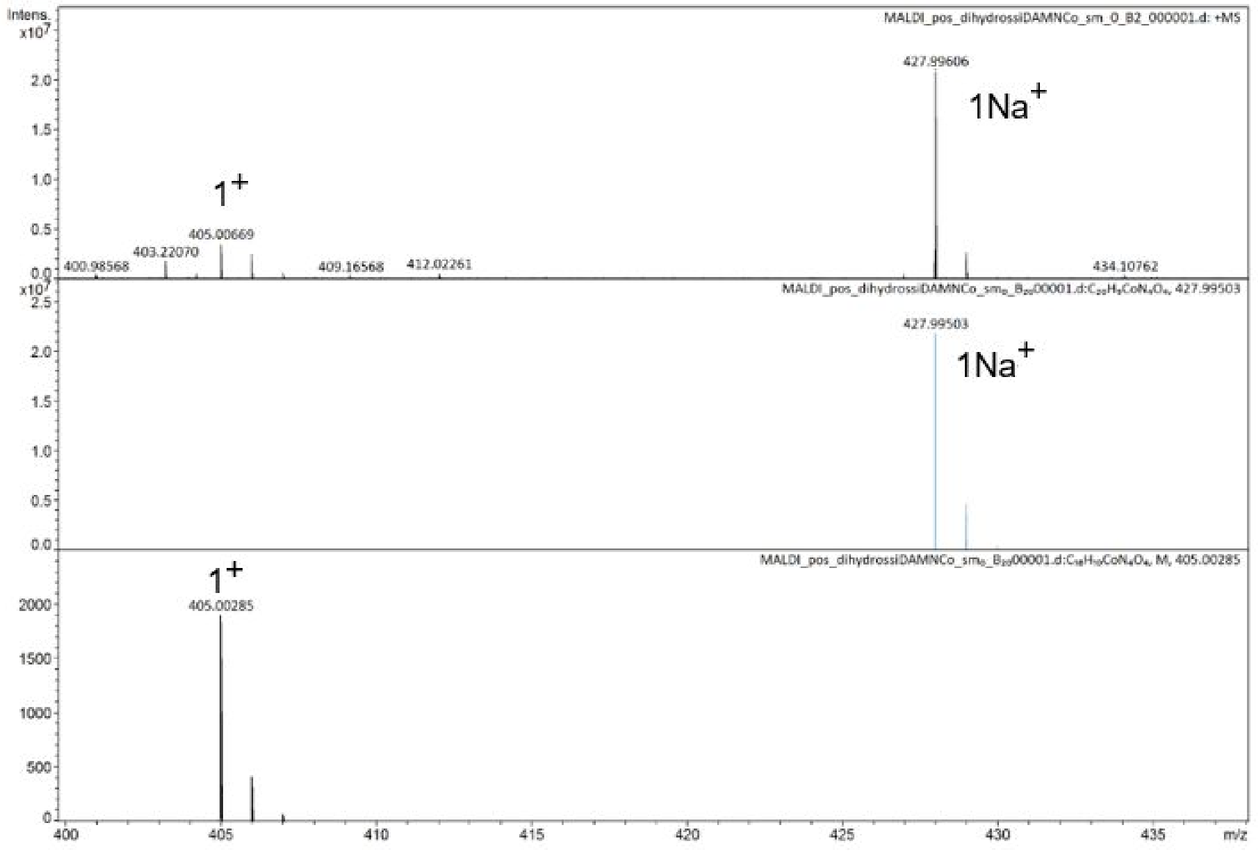

Figure S1. MALDI spectrum of complex **1** in MeOH. The upper trace is the experimental trace whereas the lowers are the theoretical ones.

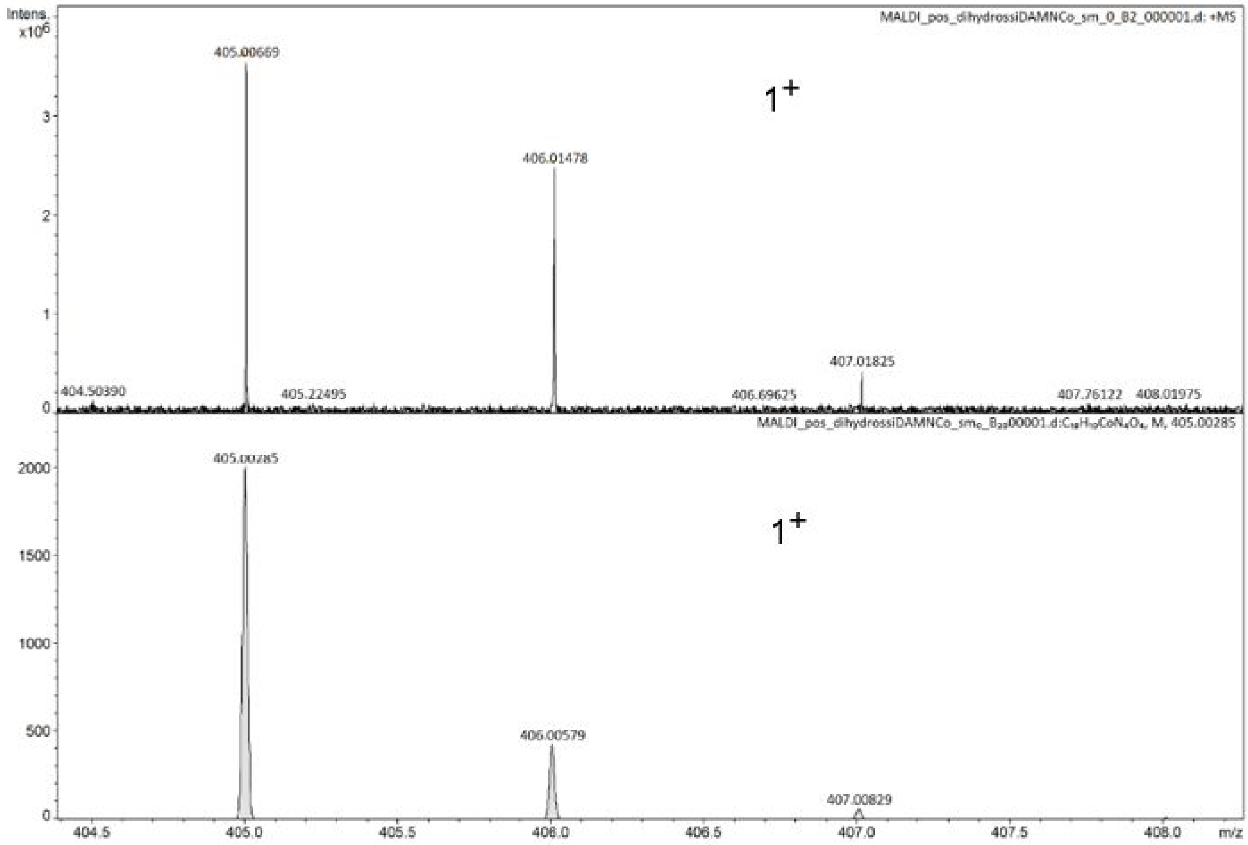

Figure S2. Enlargement of a region of the MALDI spectrum of complex **1** in MeOH. The upper trace is the experimental trace whereas the lower is the theoretical one.

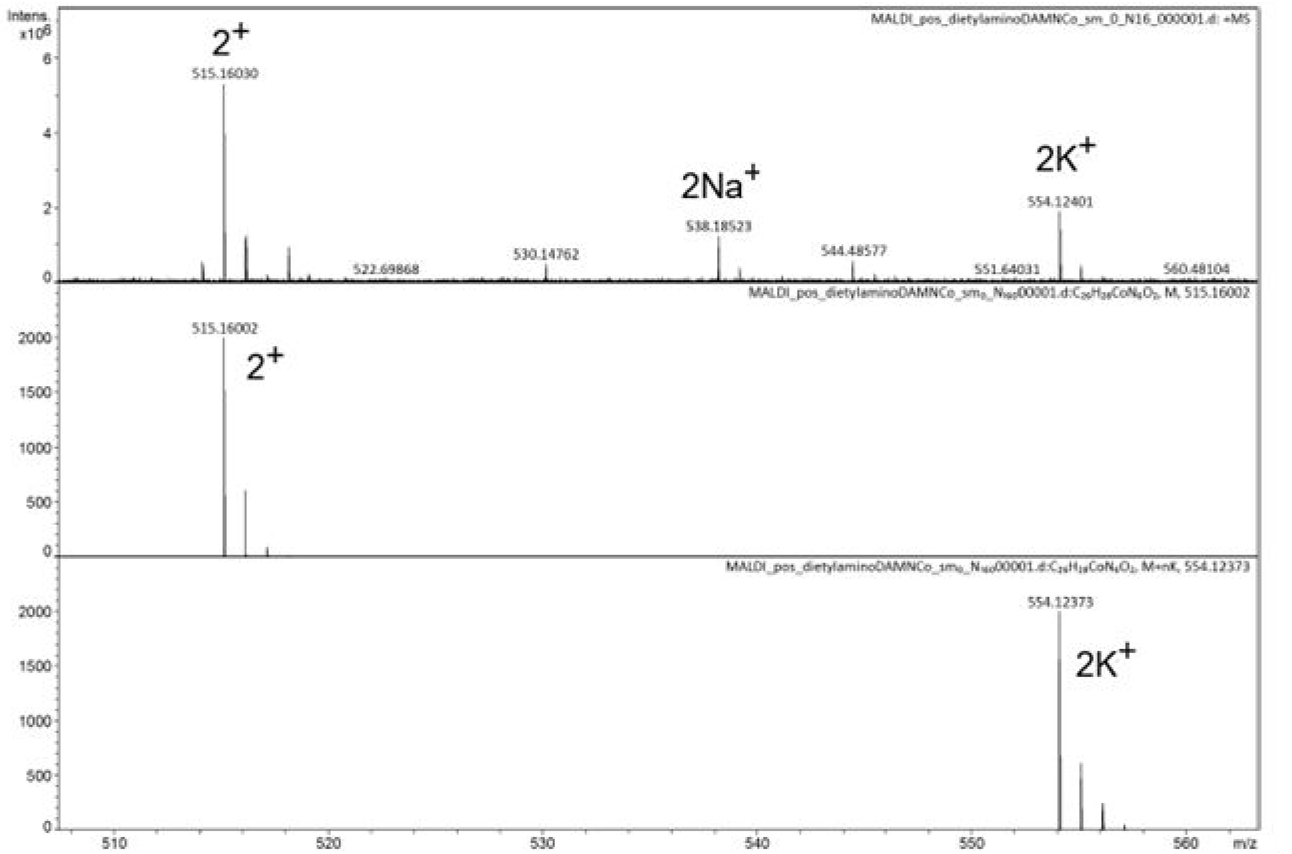

Figure S3. MALDI spectrum of complex **2** in MeOH. The upper trace is the experimental trace whereas the lower is the theoretical one.

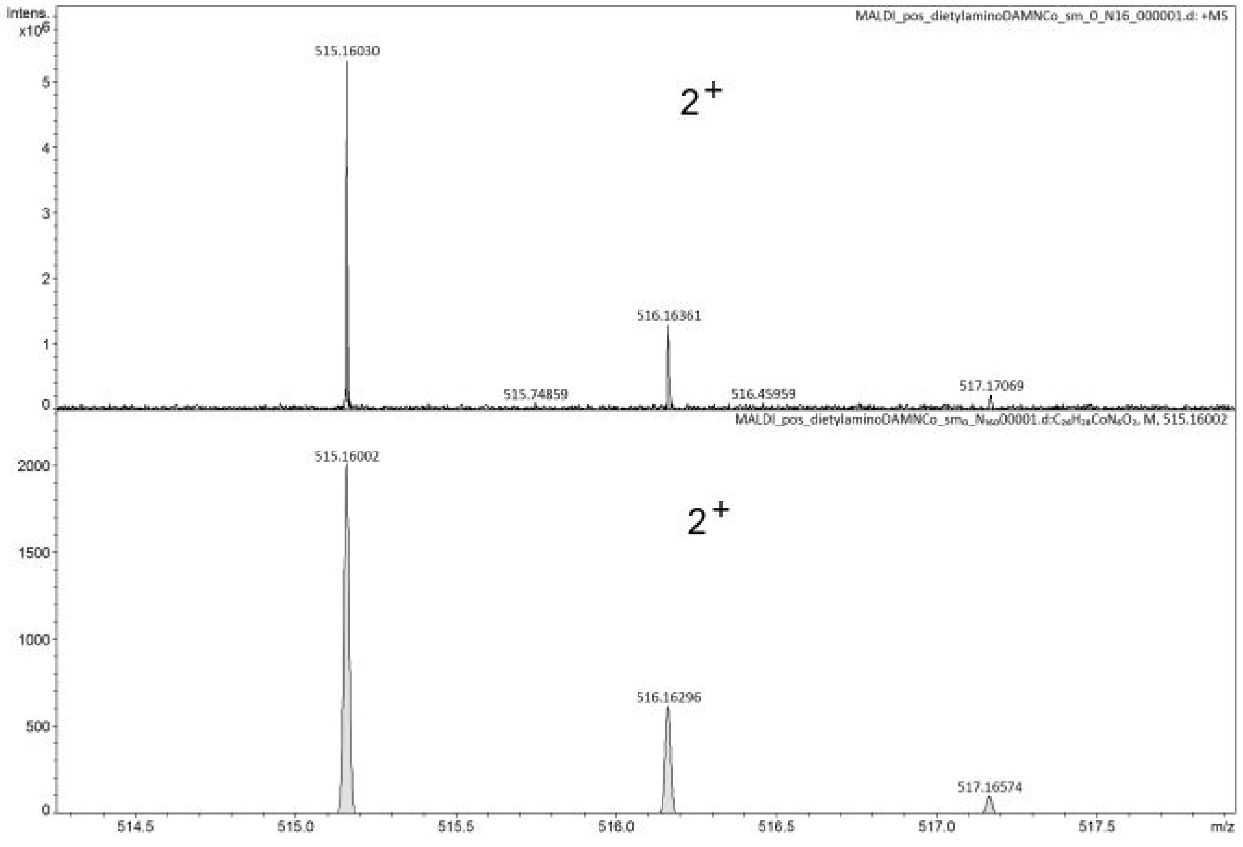

Figure S4. Enlargement of a region of the MALDI spectrum of complex **2** in MeOH. The upper trace is the experimental trace whereas the lower is the theoretical one.

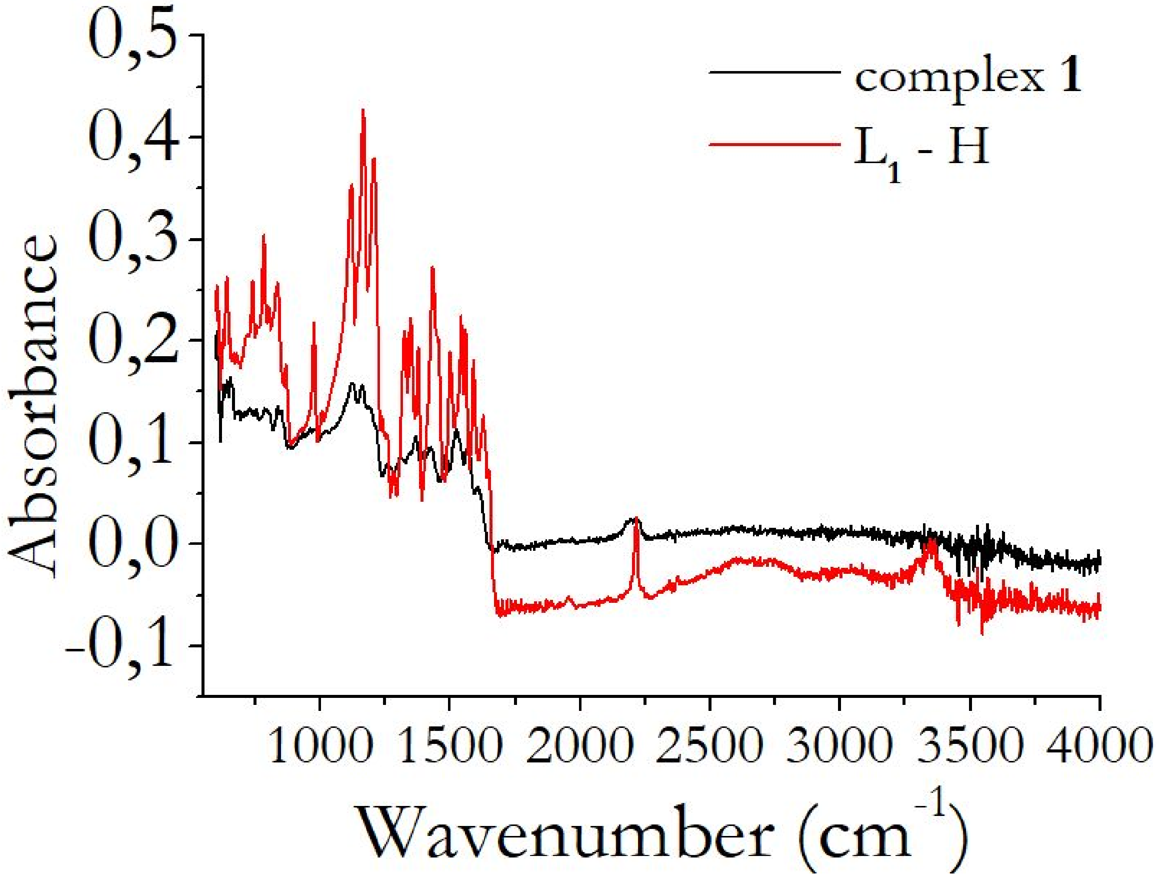

Figure S5. IR spectrum of L_1-H (red trace) and complex **1** (black trace)

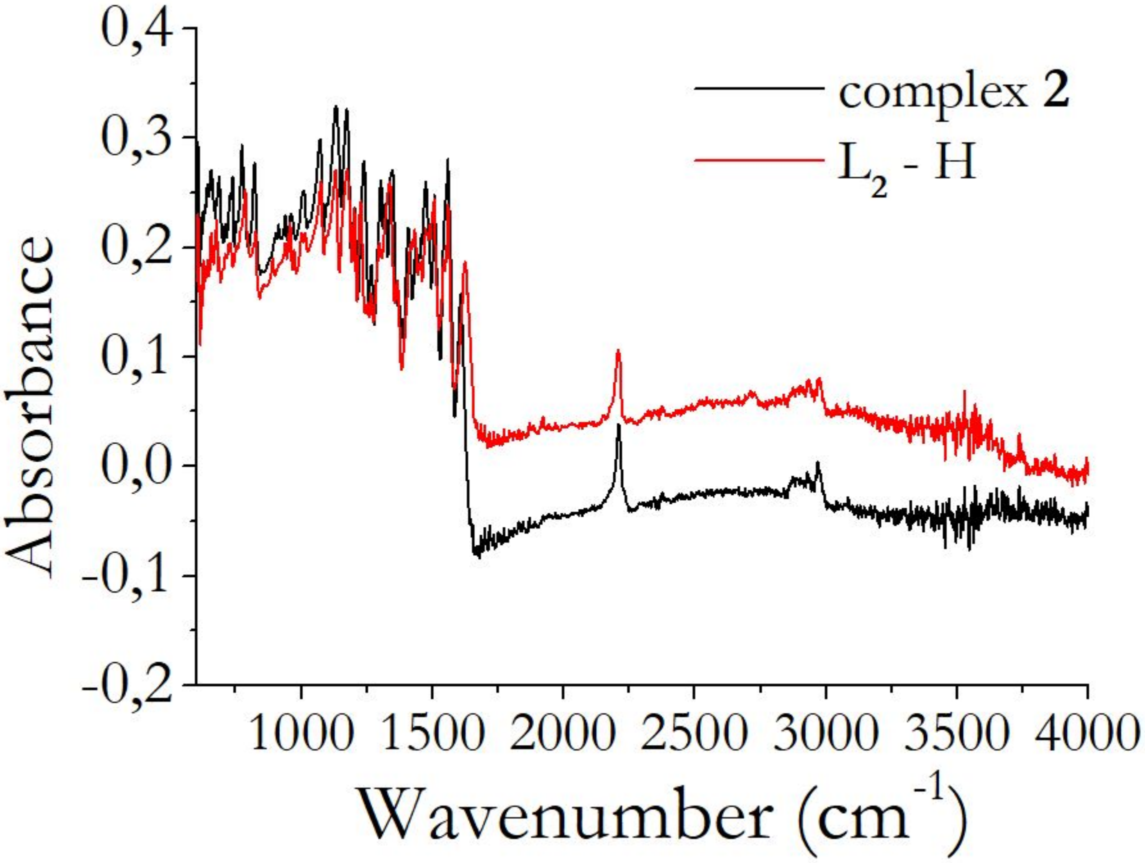

Figure S6. IR spectrum of L_2-H (red trace) and complex **2** (black trace).

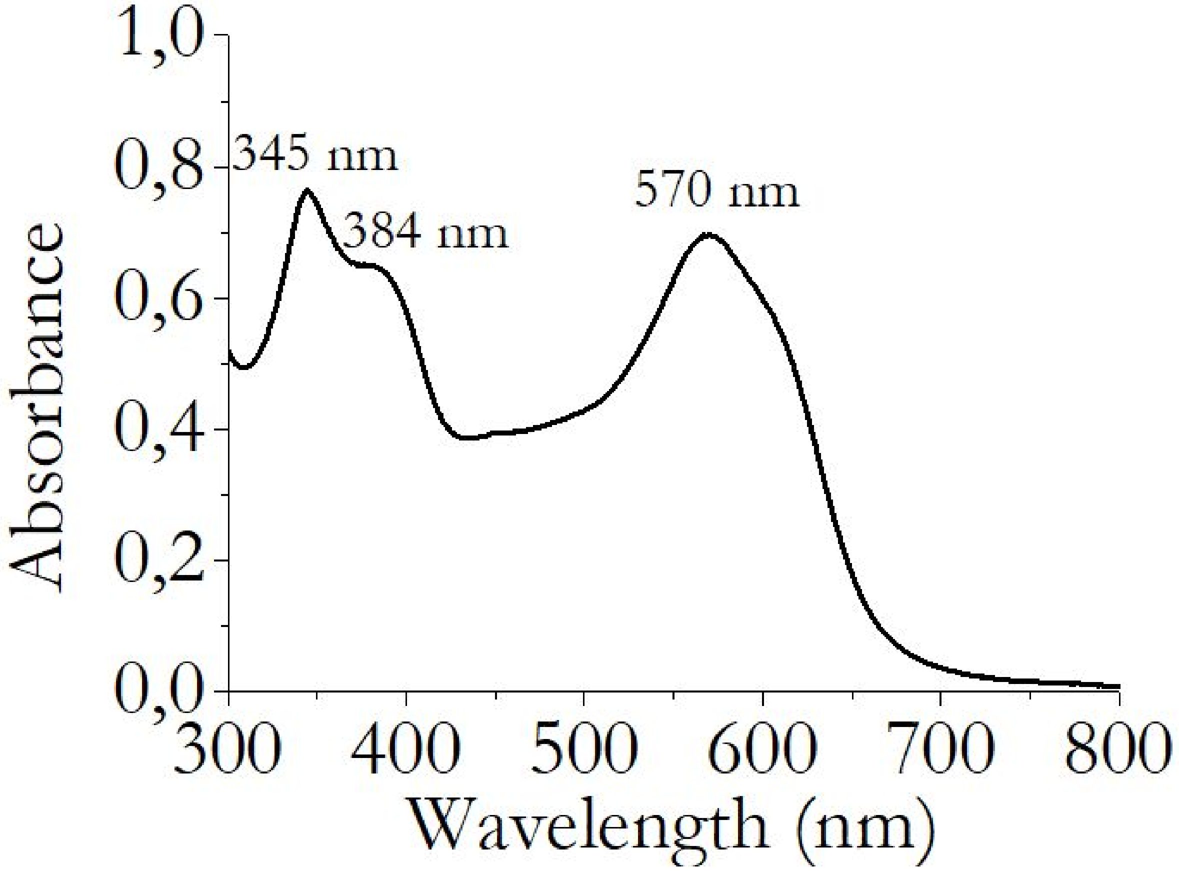

Figure S7. UV-vis spectrum of complex **1** in DMSO. [complex **1**] = 35 μM

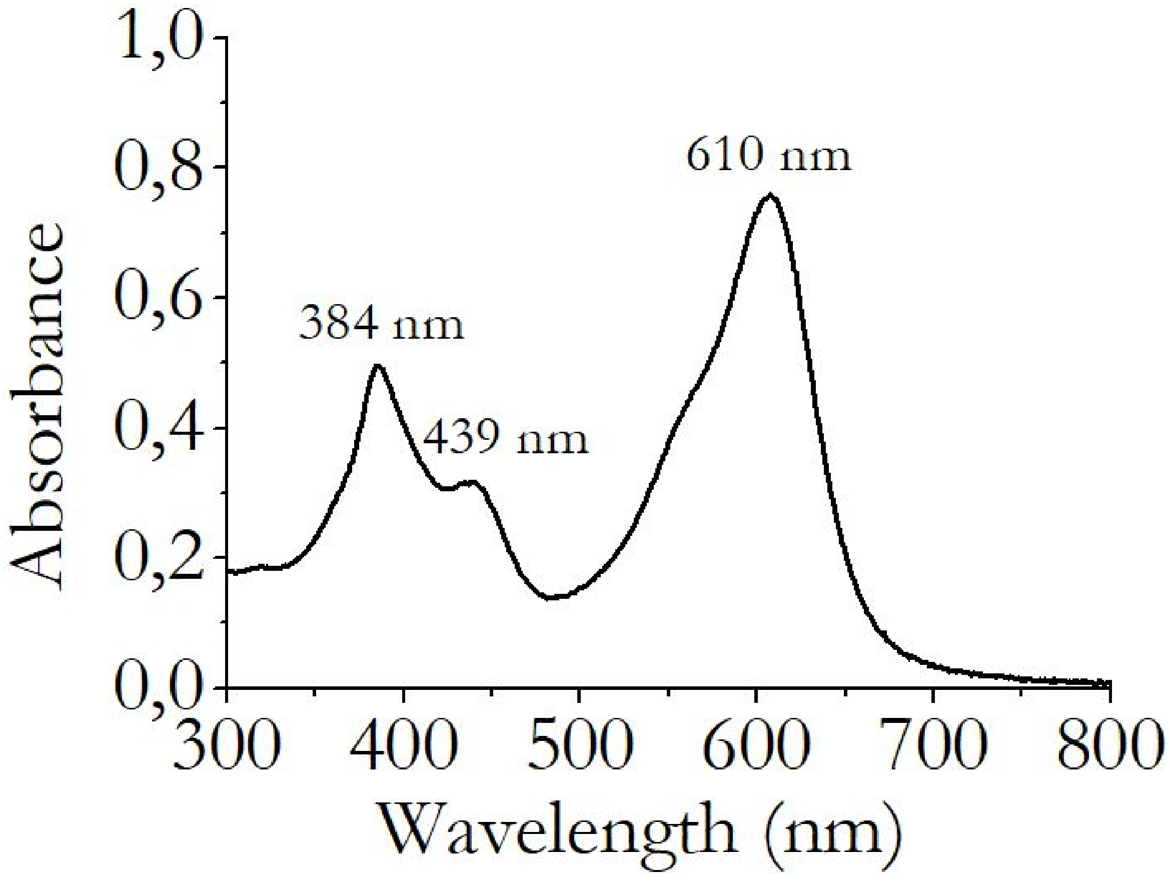

Figure S8. UV-vis spectrum of complex **2** in DMSO. [complex **2**] = 20 μM

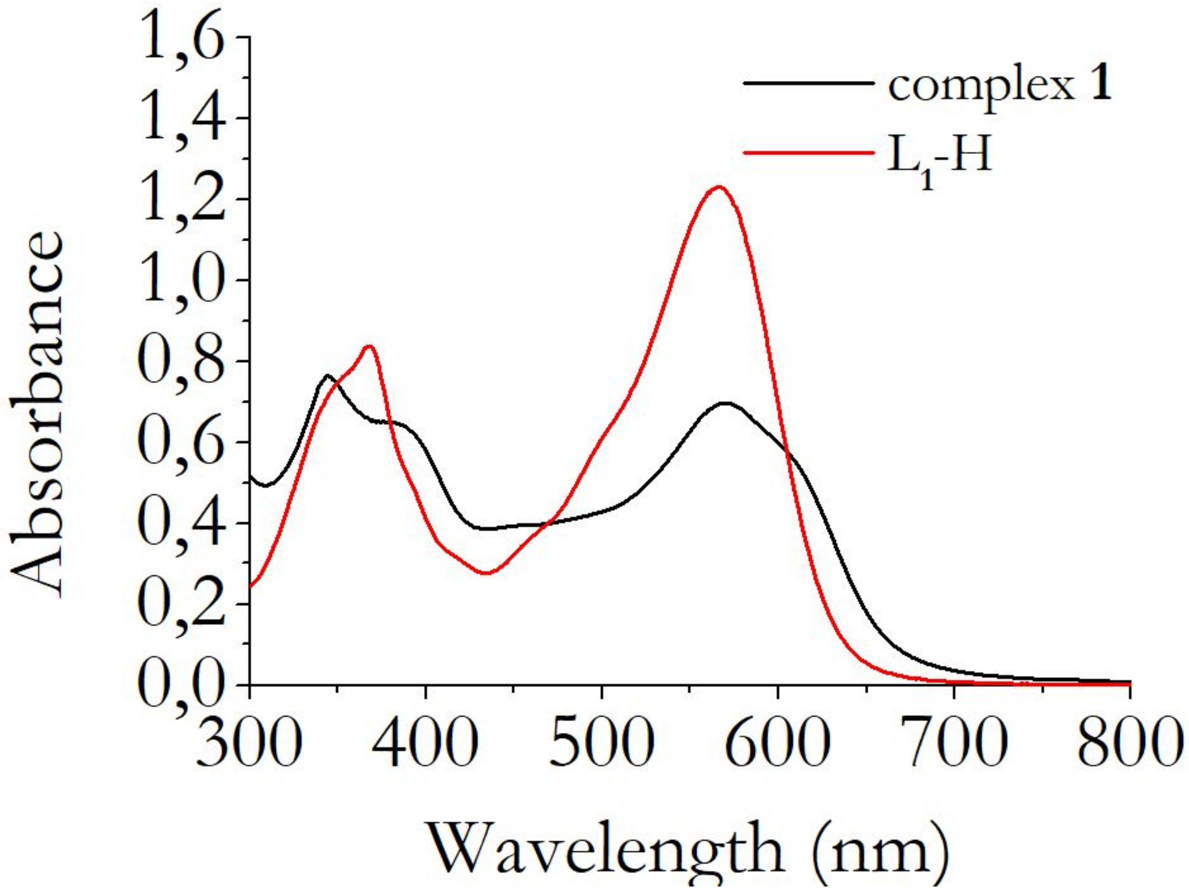

Figure S9. UV-vis spectrum of L_1-H and of complex **1** in DMSO. [L_1-H] = [complex **1**] = 35 μM

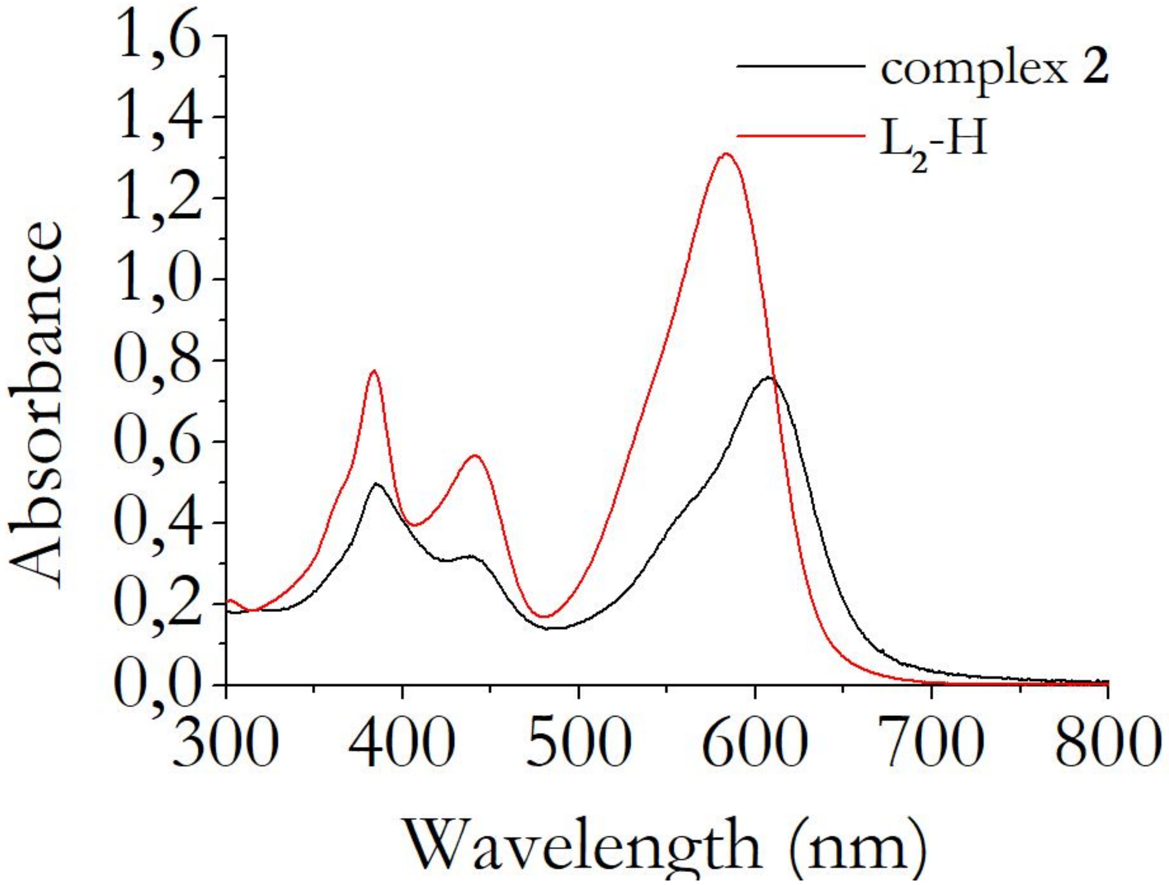

Figure S10. UV-vis spectrum of L_2-H and of complex **2** in DMSO. [L_2-H] = [complex **2**] = 20 µM

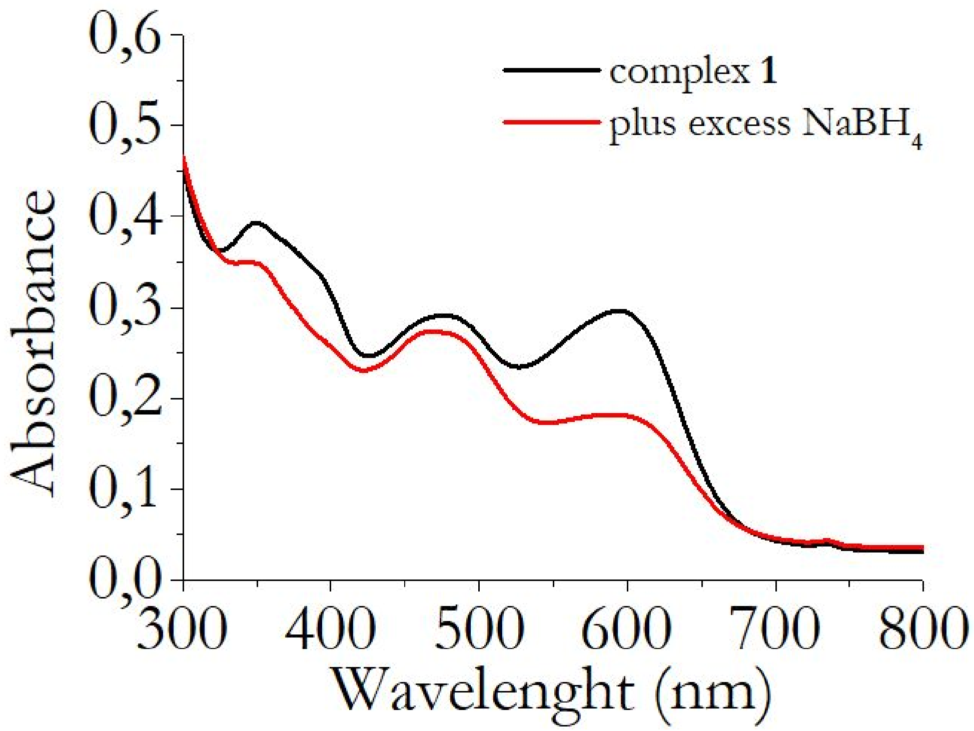

Figure S11. UV-vis spectrum of complex **1** in the presence of 10 equivalents of $NaBH_4$ in DMSO. [complex **1**] = 20 μM.

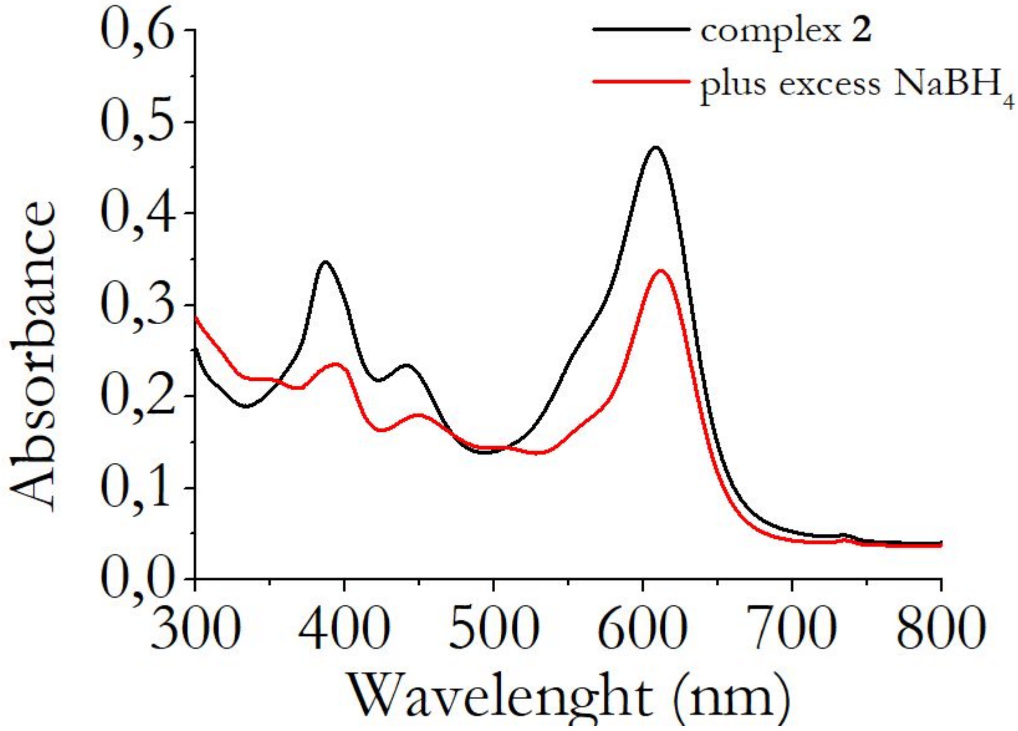

Figure S12. UV-vis spectrum of complex **2** in the presence of 10 equivalents of $NaBH_4$ in DMSO. [complex **2**] = 15 μM.

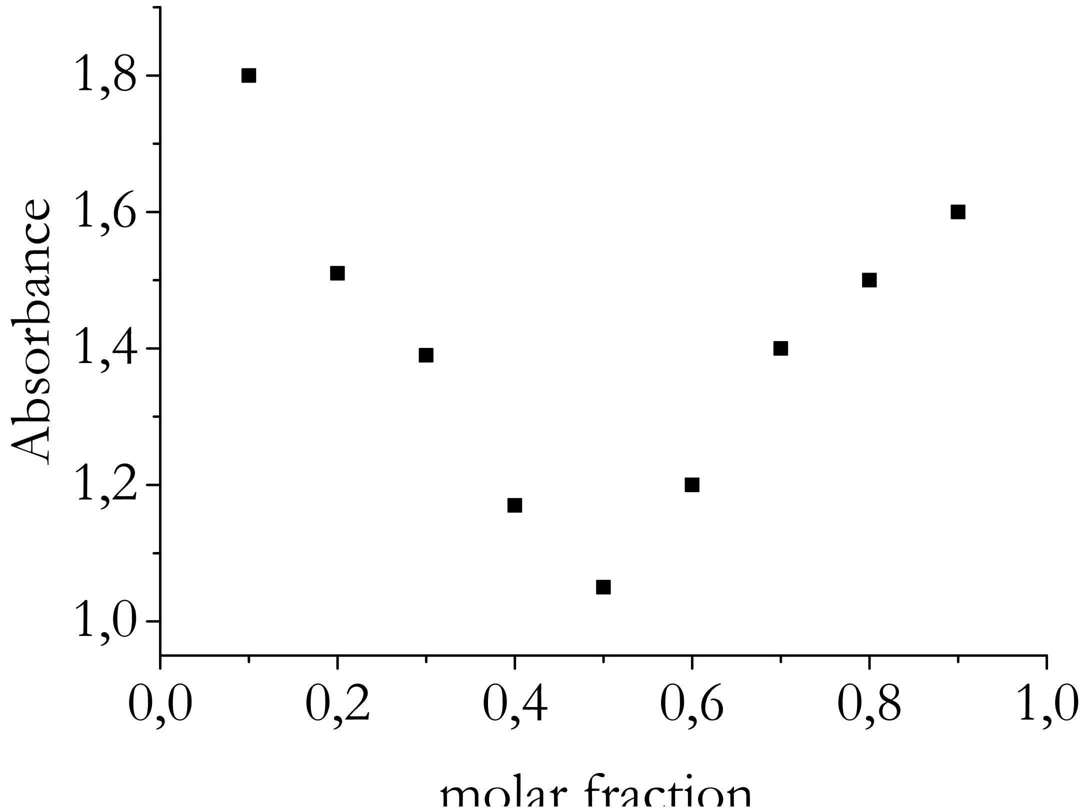

Figure S13. Job's plot (rt, DMSO), [complex **1**] + [HS^-] = 0.005 M. The absorption intensities are plotted versus the molar fraction.

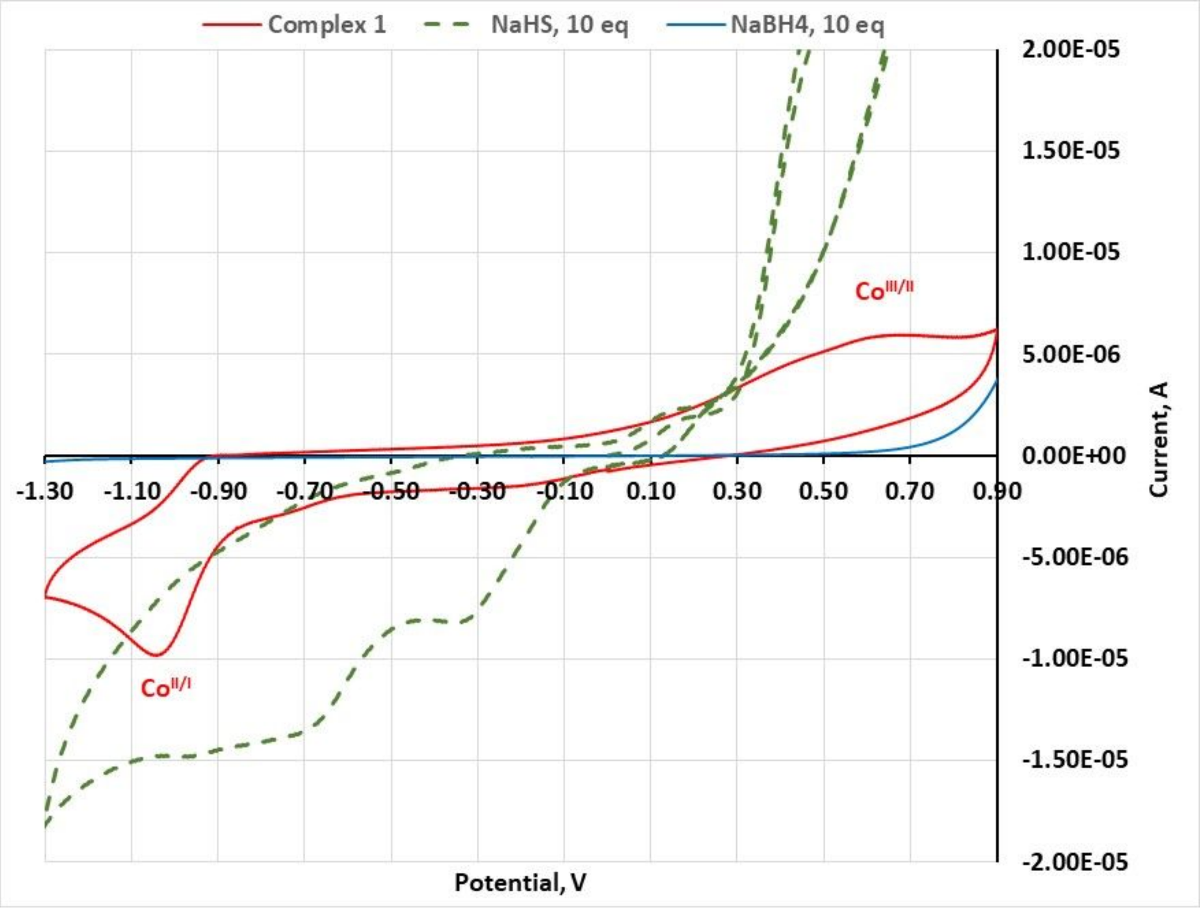

Figure S14: Cyclic voltammogram of complex **1** in the absence and presence of HS^- or $NaBH_4$.

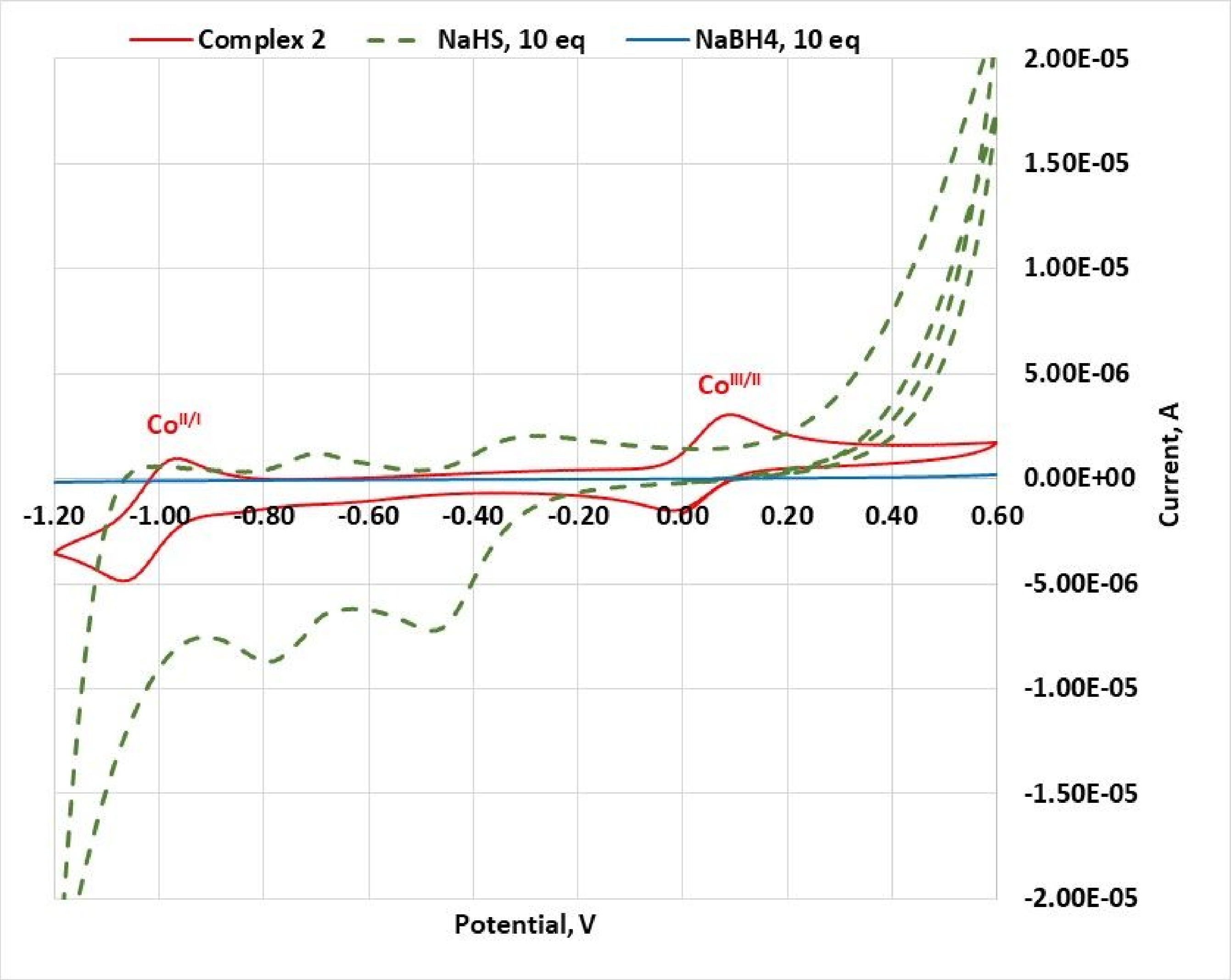

Figure S15: Cyclic voltammogram of complex **2** in the absence and presence of HS^- or $NaBH_4$.

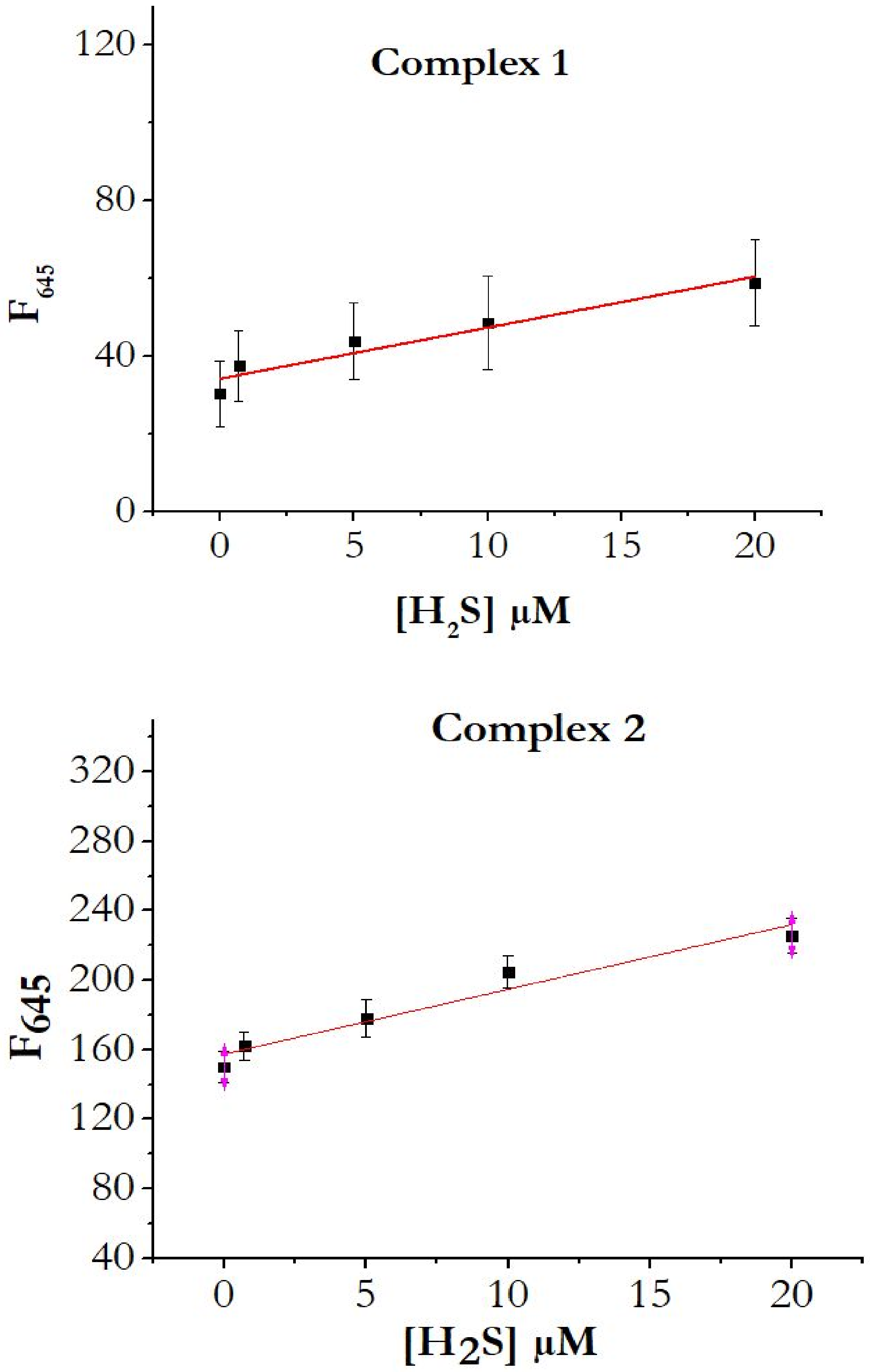

Figure S16. Calibration line of (a) complex **1** (2.0×10^{-5} M in DMSO; λ_{exc} = 570 nm; λ_{em} = 645 nm) and (b) complex **2** (2.0×10^{-5} M in DMSO; λ_{exc} = 610 nm; λ_{em} = 645 nm) for the addition of increasing amounts of HS^-; PMT voltage set to 600 V.

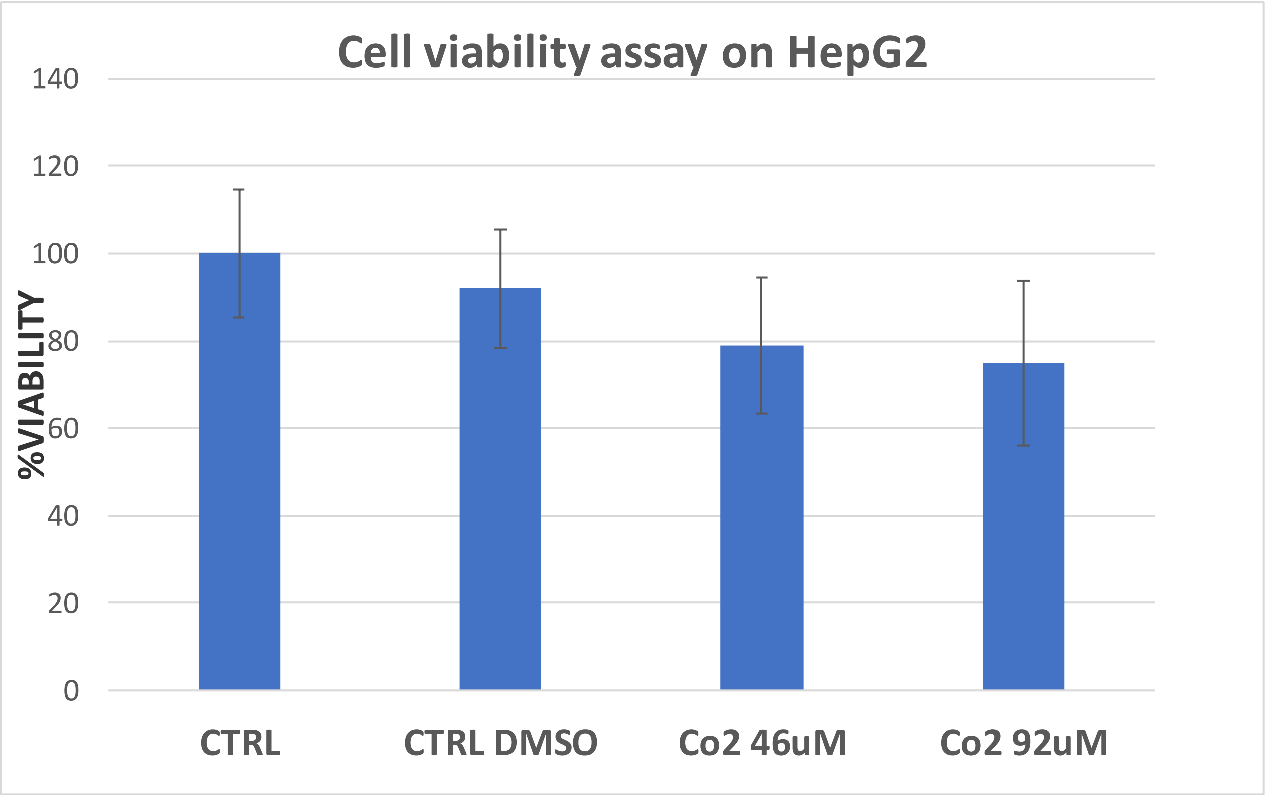

Figure S17. Cytotoxicity evaluation of complex **2** in HepG2 cell line by MTT assay. Cells were incubated 2 h with complex **2** (46µM and 92µM). Non-treated cells (Ctrl) and cells incubated with DMSO (Ctrl DMSO) were used as controls. Cell viability was compared with non-treated cells (Ctrl) and data were reported as the mean percentage ± standard deviation.

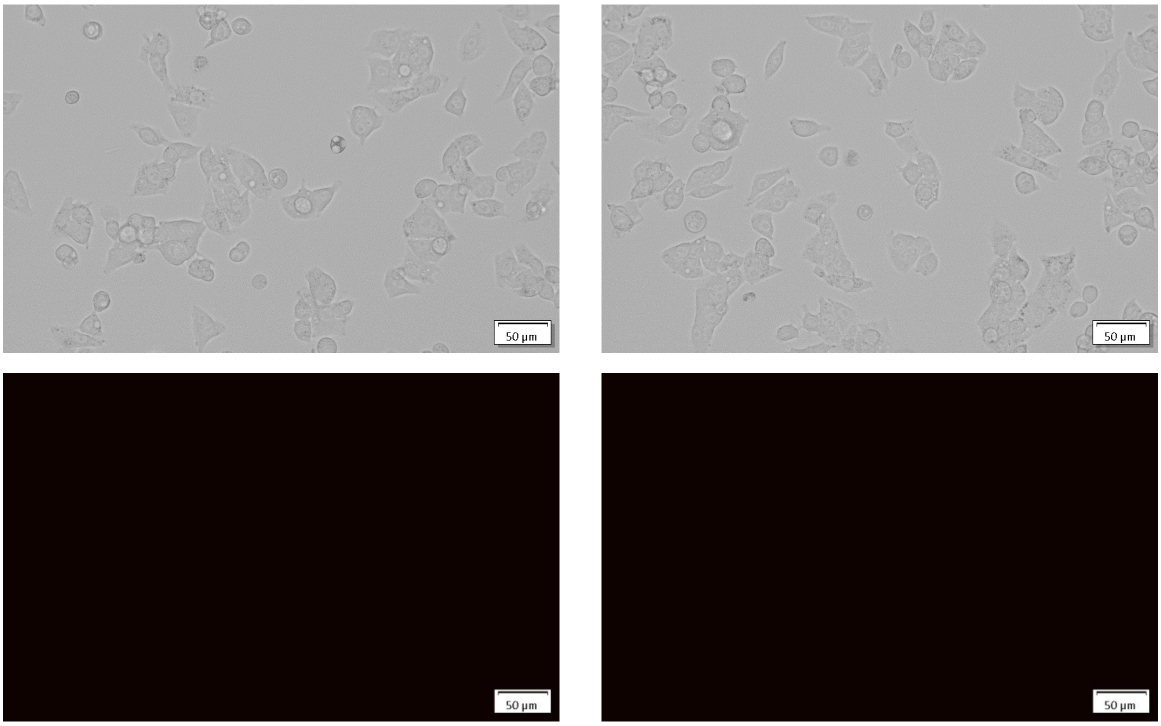

Figure S18. Fluorescence microscopy images of non-treated HepG2 cells.

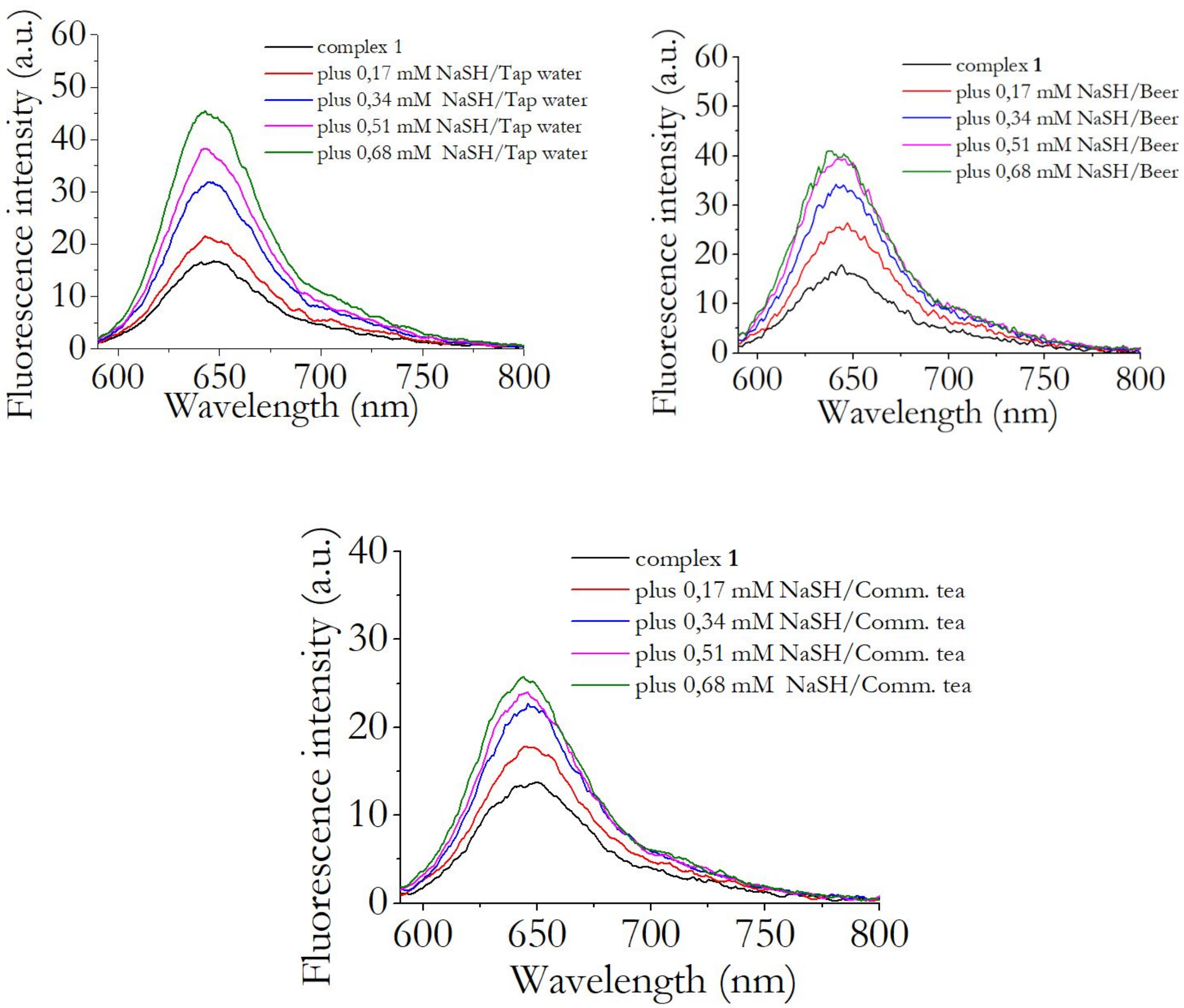

Figure S19. Emission spectra of complex **1** before and after the addition of increasing amounts of NaSH in tap water (a), in beer (b) and in commercial tea (c). [Complex] = 1×10^{-5} M; All spectra were registered in DMSO with λ_{exc} = 570 nm.

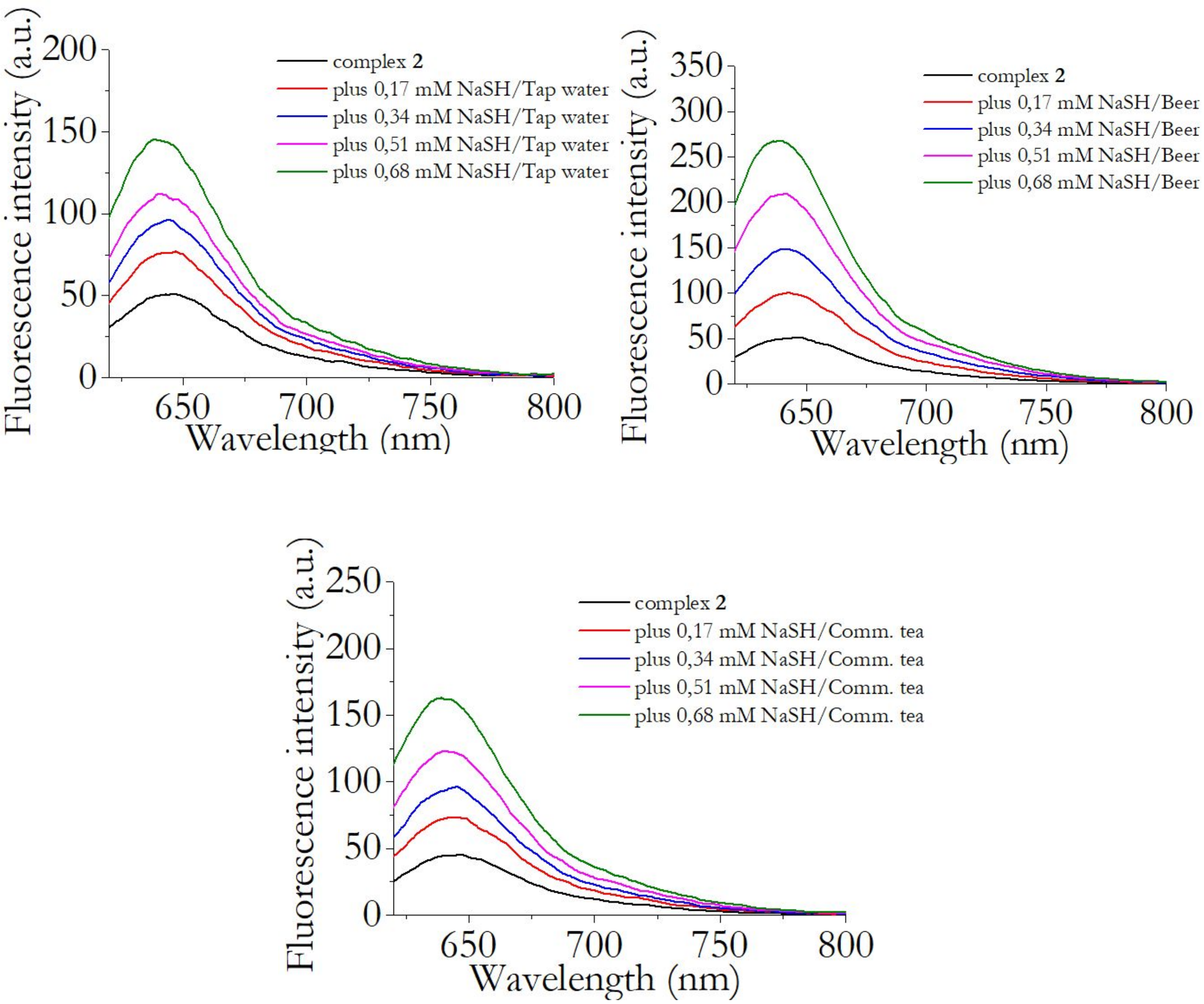

Figure S20. Emission spectra of complex **2** before and after the addition of increasing amounts of NaSH in tap water (a), in beer (b) and in commercial tea (c). [Complex] = 1 × 10^{-5} M. All spectra were registered in DMSO with λ_{exc} = 610 nm.

Table S1. Photophysical features of the title complexes.

	Φ_F	λ_a^{max}	λ_f^{max}
Complex **1**	0.06	570 nm	642 nm
Complex **2**	0.2	607 nm	640 nm

Table S2. UV-vis fitting data for the determination of the binding constant between complex 1 and HS^- *via* a Benesi-Hildebrand plot (shown at the bottom).

N	[Co], μM	$[HS^-]$, μM	eq	A^{611}, nm	ΔA	1/ΔA	1/[H°]
1	10	10	0	0	0.374	-	-
2	10	10	10	1	0.354	-96.57	0.100
3	10	10	20	2	0.335	-48.76	0.050
4	10	10	40	4	0.313	-31.45	0.025
5	10	10	60	6	0.290	-23.08	0.017
6	10	10	80	8	0.270	-19.13	0.013
8	10	10	100	10	0.253	-16.53	0.010
9	10	10	200	20	0.227	-13.23	0.005
10	10	10	300	30	0.206	-11.03	0.003
11	10	10	400	40	0.181	-9.77	0.003
12	10	10	500	50	0.159	-9.03	0.002

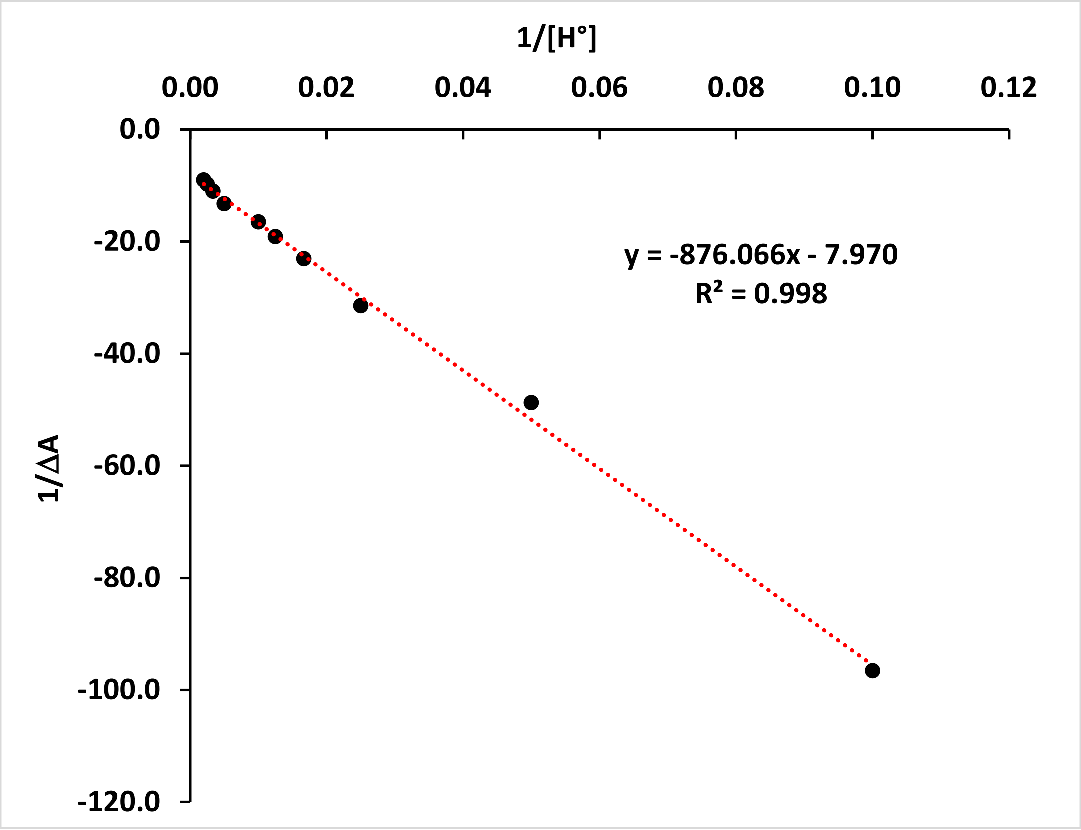

Table S3. Fluorescence fitting data for the determination of the binding constant between complex 1 and HS^- *via* a Benesi-Hildebrand plot (shown at the bottom).

N	[Co], µM	[HS^-], µM	eq	F, nm	ΔF	1/ΔF	1/[H°]
1	10	0	0	114.0	0.0	-	-
2	10	88	9	121.7	7.7	-	-
3	10	40	4	150.3	36.3	0.0276	25.000
4	10	100	10	183.0	69.0	0.0145	10.000
5	10	200	20	225.1	111.1	0.0090	5.000
6	10	350	35	237.8	123.8	0.0081	2.857
8	10	500	50	272.3	158.3	0.0063	2.000
9	10	597	60	276.1	162.1	0.0062	1.675
10	10	700	70	284.9	170.9	0.0059	1.429

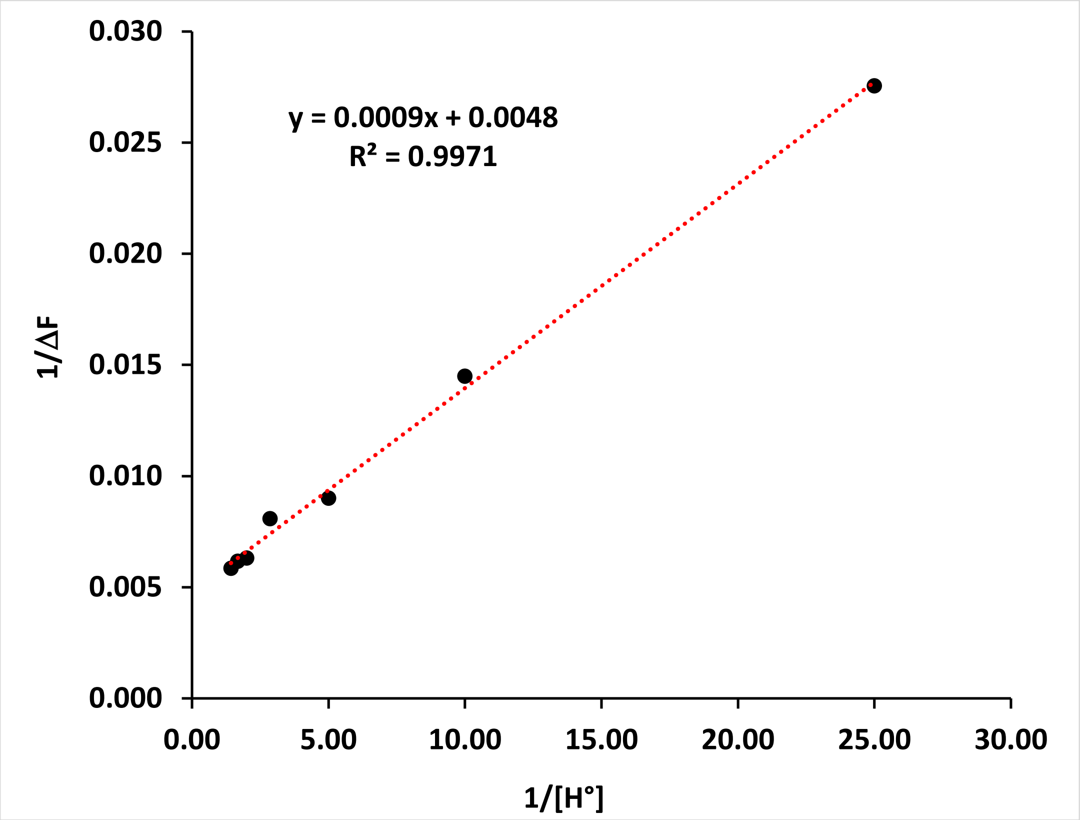

Table S4. UV-vis fitting data for the determination of the binding constant between complex 2 and HS^- *via* a Benesi-Hildebrand plot (shown at the bottom).

N	[Co], μM	[HS⁻], μM	eq	A^{611}, nm	ΔA	1/ΔA	1/[H°]
1	10	0	0	0.374	0.000	-	-
2	10	10	1	0.354	-0.019	-51.54	0.100
3	10	20	2	0.335	-0.039	-25.71	0.050
4	10	40	4	0.313	-0.061	-16.43	0.025
5	10	60	6	0.290	-0.084	-11.91	0.017
6	10	80	8	0.270	-0.103	-9.68	0.013
7	10	100	10	0.253	-0.121	-8.24	0.010
8	10	200	20	0.227	-0.147	-6.80	0.005
9	10	300	30	0.206	-0.168	-5.97	0.003
10	10	400	40	0.181	-0.192	-5.20	0.003
11	10	500	50	0.159	-0.215	-4.64	0.002

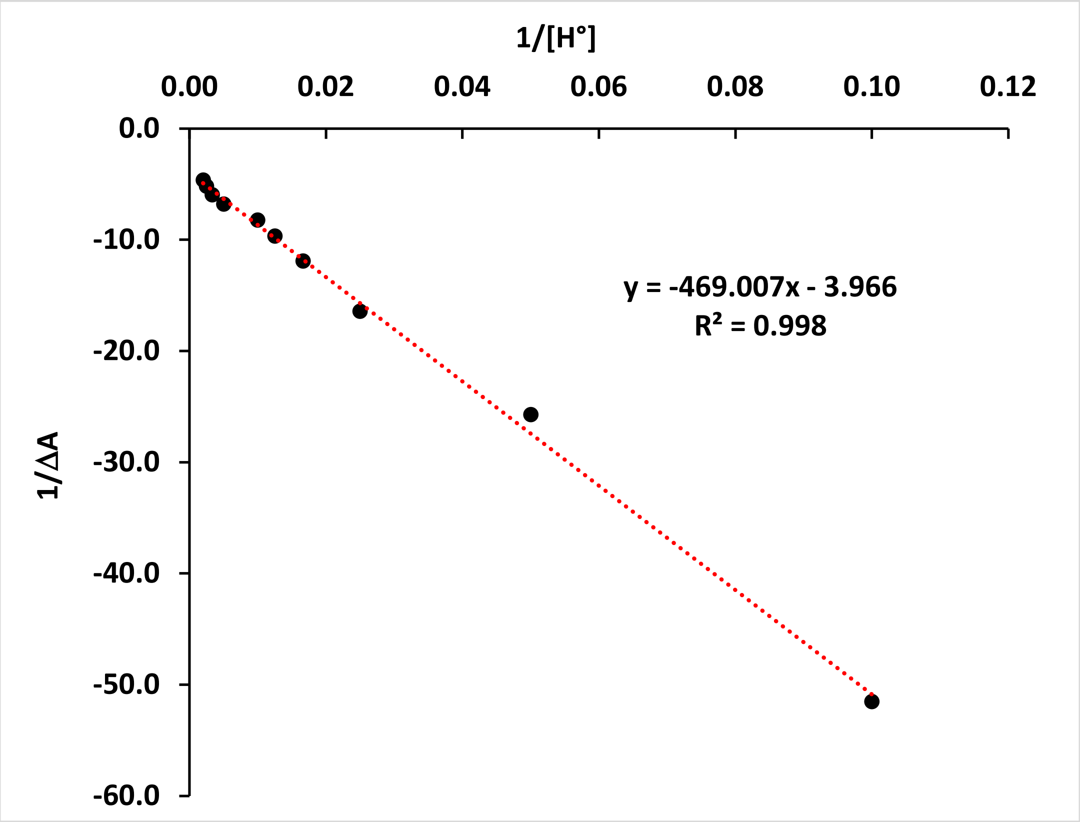

Table S5. Fluorescence fitting data for the determination of the binding constant between complex 2 and HS^- *via* a Benesi-Hildebrand plot (shown at the bottom).

N	[Co], µM	[HS⁻], µM	eq	F, nm	ΔF	1/ΔF	1/[H°]
1	10	0	0	114.0	0.0	-	-
2	10	88	9	121.7	7.7	-	-
3	10	40	4	150.3	36.3	0.0276	25.000
4	10	100	10	183.0	69.0	0.0145	10.000
5	10	200	20	225.1	111.1	0.0090	5.000
6	10	350	35	237.8	123.8	0.0081	2.857
7	10	500	50	272.3	158.3	0.0063	2.000
8	10	597	60	276.1	162.1	0.0062	1.675
9	10	700	70	284.9	170.9	0.0059	1.429

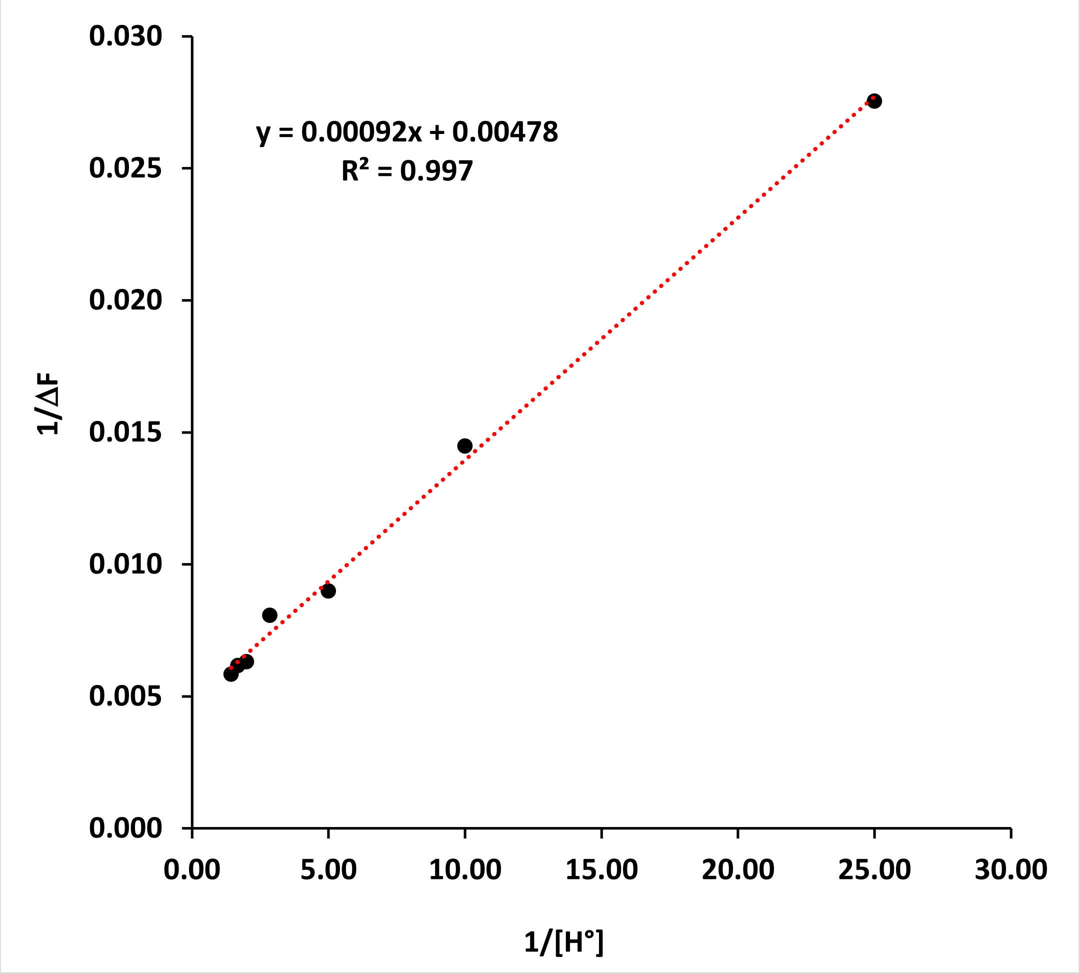

Table S6. The standard deviation of the blank (σ), the absolute value of the slope of the calibration line (K) (Figure S16) and LOD values.

**Complex**	**σ**	**K**	**LOD (µM)**
**1**	1	1.3	3.03 µM
**2**	3	3.7	2.60 µM
